# Distinct populations of inflammatory fibroblasts and myofibroblasts in pancreatic cancer

**DOI:** 10.1084/jem.20162024

**Published:** 2017-03-06

**Authors:** Daniel Öhlund, Abram Handly-Santana, Giulia Biffi, Ela Elyada, Ana S. Almeida, Mariano Ponz-Sarvise, Vincenzo Corbo, Tobiloba E. Oni, Stephen A. Hearn, Eun Jung Lee, Iok In Christine Chio, Chang-Il Hwang, Hervé Tiriac, Lindsey A. Baker, Dannielle D. Engle, Christine Feig, Anne Kultti, Mikala Egeblad, Douglas T. Fearon, James M. Crawford, Hans Clevers, Youngkyu Park, David A. Tuveson

**Affiliations:** 1 Cold Spring Harbor Laboratory, Cold Spring Harbor, NY 11724; 2 Lustgarten Foundation Pancreatic Cancer Research Laboratory, Cold Spring Harbor, NY 11724; 3 Department of Surgical and Perioperative Sciences, Surgery, Umeå University, 901 85 Umeå, Sweden; 4 APC Microbiome Institute and School of Microbiology, University College Cork, Cork, Ireland; 5 Department of Oncology, Clinica Universidad de Navarra, CIMA, IDISNA, Pamplona 31008, Spain; 6 ARC-Net centre for applied research on cancer, University and Hospital Trust of Verona, 37134 Verona, Italy; 7 Department of Diagnostic and Public Health, University and Hospital Trust of Verona, 37134 Verona, Italy; 8 Graduate Program in Molecular and Cellular Biology, Stony Brook University, Stony Brook, NY 11794; 9 University of Cambridge, Cancer Research UK, Cambridge Institute, Cambridge, UK; 10 Hofstra Northwell School of Medicine, Hempstead, NY 11550; 11 Hubrecht Institute, Royal Netherlands Academy of Arts and Sciences (KNAW), University Medical Centre Utrecht and CancerGenomics.nl, 3584 CT Utrecht, Netherlands

## Abstract

Pancreatic stellate cells (PSCs) differentiate into cancer-associated fibroblasts (CAFs) that produce desmoplastic stroma, thereby modulating disease progression and therapeutic response in pancreatic ductal adenocarcinoma (PDA). However, it is unknown whether CAFs uniformly carry out these tasks or if subtypes of CAFs with distinct phenotypes in PDA exist. We identified a CAF subpopulation with elevated expression of α-smooth muscle actin (αSMA) located immediately adjacent to neoplastic cells in mouse and human PDA tissue. We recapitulated this finding in co-cultures of murine PSCs and PDA organoids, and demonstrated that organoid-activated CAFs produced desmoplastic stroma. The co-cultures showed cooperative interactions and revealed another distinct subpopulation of CAFs, located more distantly from neoplastic cells, which lacked elevated αSMA expression and instead secreted IL6 and additional inflammatory mediators. These findings were corroborated in mouse and human PDA tissue, providing direct evidence for CAF heterogeneity in PDA tumor biology with implications for disease etiology and therapeutic development.

## Abstract (precis)

Öhlund et al. develop a three-dimensional co-culture platform of neoplastic pancreatic ductal organoids and pancreatic stellate cells (PSCs) to characterize the dynamic crosstalk between cancer cells and stromal cells, and to address stromal heterogeneity. The co-cultures reveal the co-existence of two phenotypically distinct populations of PSCs, providing insights into PDA biology and prompting a reconsideration of interventional strategies.

## Introduction

Pancreatic ductal adenocarcinoma (PDA) has one of the worst outcomes among all cancers, with a median survival of ∼6 mo and a 5-yr survival rate of <8% (Siegel et al., 2016). Patients are often diagnosed late during disease progression, when curative surgical approaches are not feasible. Indeed, the current systemic therapies for patients with advanced PDA provide only temporary benefits, highlighting the need for new therapeutic strategies.

PDA is characterized by abundant desmoplasia that constitutes up to 90% of the total tumor volume and contains extracellular matrix (ECM), immune cells, vasculature, and cancer-associated fibroblasts (CAFs; Moir et al., 2015). CAFs secrete ECM and soluble factors that stimulate cancer progression, and are believed to be derived from mesenchymal cells of different origins that are resident or recruited to the pancreas by neoplastic cells (Öhlund et al., 2014; Moffitt et al., 2015; Kalluri, 2016). A major source of CAFs in PDA is pancreatic stellate cells (PSCs), which are resident mesenchymal cells of the pancreas that store lipid droplets and express fibroblast-activation protein α (FAP; Bachem et al., 2005; Erkan et al., 2012; Apte et al., 2013; Moir et al., 2015). Upon activation, PSCs express the myofibroblast protein α-smooth muscle actin (αSMA, gene name *Acta2*) and secrete factors that stimulate tumor growth, cell survival, and metastasis (Bachem et al., 2005; Hwang et al., 2008; Vonlaufen et al., 2008; Xu et al., 2010). PSCs are also reported to produce the majority of ECM in PDA (Apte et al., 2004), which acts as a physical barrier that impairs drug delivery (Olive et al., 2009; Jacobetz et al., 2012; Provenzano et al., 2012), and may also biochemically contribute to drug resistance (Garrido-Laguna et al., 2011; Straussman et al., 2012).

Attempts to target the stroma, either by directly targeting CAFs (Olive et al., 2009; Froeling et al., 2011; Sherman et al., 2014), or by enzymatically digesting the ECM (Jacobetz et al., 2012; Provenzano et al., 2012), have resulted in reduced tumor growth and improved response to chemotherapy in mouse models and patients (Hingorani et al., 2016). These preclinical and clinical findings have nominated the PDA stroma, particularly CAFs, as attractive targets for drug development. However, several recent reports have questioned the role of CAFs in PDA maintenance (Bijlsma and van Laarhoven, 2015). Genetic disruption or prolonged pharmacological inhibition of sonic hedgehog, a ligand that stimulates CAFs (Tian et al., 2009; Lee et al., 2014; Rhim et al., 2014), or depletion of αSMA-expressing cells (Özdemir et al., 2014), resulted in undifferentiated PDA tumors and decreased survival in mice. Furthermore, clinical trials of Smoothened inhibitors, which targeted the G protein–coupled receptor downstream of hedgehog signaling, have failed to demonstrate benefits for PDA patients (Kim et al., 2014), with one randomized trial reporting adverse effects (Business Wire, 2012). Although these findings demonstrate the need to use caution when targeting CAFs, they also highlight the need to systematically determine the composition and function of the PDA stroma to improve the development of effective stroma-targeting drugs. Indeed, based on the expression patterns of various fibroblast markers in vivo, evidence is emerging on the existence of different subtypes of CAFs (Sugimoto et al., 2006; Öhlund et al., 2014; Kalluri, 2016). However, no precise characterization of CAF subtypes has been performed. Here, we investigate CAF heterogeneity in a novel three-dimensional co-culture system that recapitulates the in vivo symbiotic interactions of CAFs and cancer cells. Our study reveals two spatially separated, mutually exclusive, dynamic, and phenotypically distinct CAF subtypes, underscoring the stromal heterogeneity in PDA and providing an opportunity to develop agents that target specific CAF populations.

## Results and discussion

### Heterogeneous distribution of myofibroblastic CAFs in PDA

**Figure 1.**
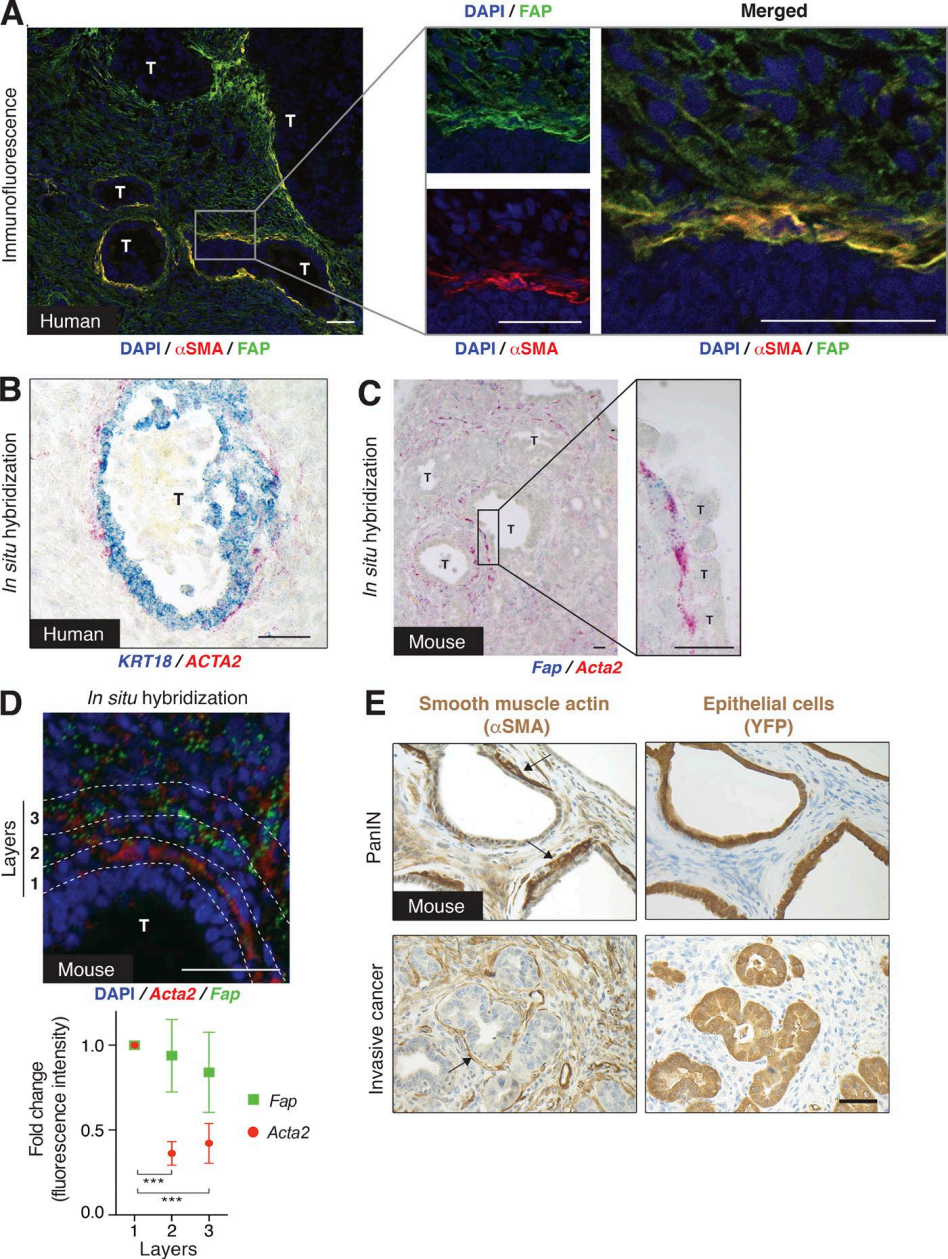
**High expression of αSMA is a distinctive property of periglandular CAFs in mouse and human PDA.** (A, left) Representative immunofluorescence (IF) co-staining of FAP (green) and αSMA (red) in a well-differentiated human PDA (*n* = 4). Counterstain, DAPI (blue). (right) Higher magnification illustrating the distribution and co-localization of FAP and αSMA. Bars, 50 µm. T, tumor glands. (B) Representative image of RNA ISH for Cytokeratin 18 (*KRT18,* blue) and αSMA (*ACTA2*, red) transcripts in a well-differentiated human PDA (*n* = 3). Bar, 50 µm. T, tumor gland. (C, left) Representative image of RNA ISH for *Fap* (blue) and *Acta2* (red) in a KPC mouse tumor (*n* = 3). (right) Higher magnification. Bars, 25 µm. T, tumor glands. (D, top) Representative image of fluorescent RNA ISH for *Fap* (green) and *Acta2* (red) in a KPC mouse tumor (*n* = 3), showing transcript distribution across three cell layers of the stroma, starting from the first layer adjacent to the tumor gland (T) and moving outwards. Counterstain, DAPI (blue). Bar, 50 µm. (bottom) Quantification of *Fap* and *Acta2* fluorescence intensity in the three cell layers. Results show mean ± SD of three tumor glands. Data are normalized to layer 1. ***, P < 0.001, unpaired Student’s *t* test. (E) Representative images of IHC of αSMA and YFP in sequential tissue sections from KPCY mice, with either preinvasive Pancreatic Intraepithelial Neoplasia (PanIN) or invasive cancer (*n* = 2). Arrows indicate areas of myCAFs. Bar, 50 µm.

To investigate the inherent heterogeneity of fibroblasts in pancreatic cancer, we evaluated the spatial distribution of αSMA, a hallmark of myofibroblasts (Desmoulière et al., 2004), in human pancreatic tumors. Immunofluorescence analysis of FAP, a PSC marker, and αSMA expression in human PDA tissues revealed that the majority of fibroblasts expressed FAP and low levels of αSMA, whereas a subpopulation of FAP^+^ cells showed substantially elevated expression levels of αSMA (Fig. 1 A). These FAP^+^ αSMA^high^ cells could also be delineated by RNA in situ hybridization (ISH), and were located in direct proximity to neoplastic cells, forming a periglandular ring surrounding cancer cell clusters (Fig. 1 B). A gradient of αSMA expression was similarly observed in tumors from KPC (*Kras^LSL-G12D/+^; Trp53^LSL-R172H/+^; Pdx-1-Cre*) mice (Fig. 1, C and D), a mouse model that recapitulates the human disease (Hingorani et al., 2005). As a result of the selective high expression of αSMA, we refer to these periglandular FAP^+^ αSMA^high^ fibroblasts as myofibroblastic CAFs (myCAFs).

To exclude the possibility that myCAFs are neoplastic cells that have undergone epithelial-to-mesenchymal transition (EMT), we analyzed tissues of a KPCY mouse model, in which all neoplastic pancreatic cells express yellow fluorescent protein (YFP; Rhim et al., 2012). Periglandular cells expressing high αSMA levels did not coexpress YFP, confirming that myCAFs are of a nonneoplastic origin (Fig. 1 E). These findings identify myCAFs as a distinct subpopulation of CAFs with a unique spatial distribution pattern in PDA.

### A novel three-dimensional co-culture platform recapitulates in vivo CAF heterogeneity

To further characterize CAFs in PDA, we studied PSCs, which are believed to be a major source of FAP^+^ CAFs in PDA stroma (Apte et al., 2004; Erkan et al., 2012). Quiescent and lipid-storing PSCs were isolated from WT C57BL/6J mice pancreata (Fig. S1, A and B), and cultured as primary cells or after immortalization with the SV40 large T Antigen. When PSCs are grown in monolayers, they lose their lipid droplets and assume a myofibroblastic phenotype indicated by αSMA expression, but can be reversed back to a quiescence and lipid-storing phenotype if embedded in Matrigel (Jesnowski et al., 2005). To verify that the characteristic phenotypes of PSCs remained after isolation and immortalization, we cultured PSCs in Matrigel and used Oil Red-O staining to confirm that they reacquired lipid droplets (Fig. S1 C). Additionally, we found that the PSCs, when cultured as a monolayer, showed an adequate response to the vitamin D analogue Calcipotriol (Sherman et al., 2014; Fig. S1 D). Moreover, addition of recombinant TGFβ to quiescent PSCs cultured in Matrigel induced the expression of TGFβ target genes, such as *Ctgf* and *Col1a1* (Fig. S1 E), demonstrating that the isolated PSCs still respond to common stromal cues.

**Figure 2.**
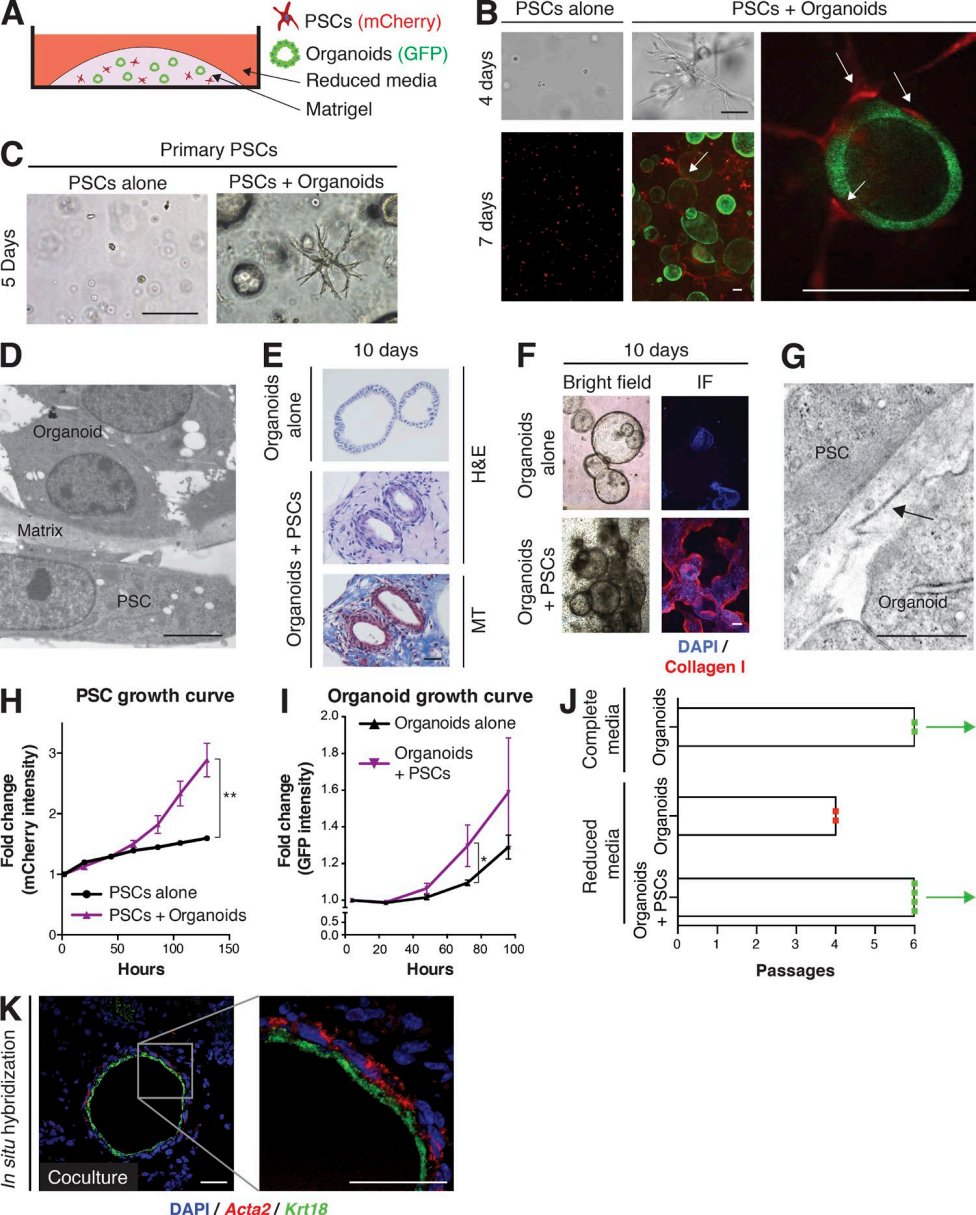
**Co-cultures of mouse PSCs and pancreatic cancer organoids recapitulate properties of PDA desmoplasia.** (A) Schematic illustration of the co-culture platform. (B) Representative images of mCherry-labeled PSCs (red) cultured alone or in co-culture with GFP-labeled tumor-derived organoids (green), and imaged by confocal microscopy after 4 or 7 d in bright field and by fluorescent microscopy (*n* = 3). Arrows point to close interactions between organoids and PSCs. Bars, 100 µm. (C) Bright field images of primary PSCs plated in Matrigel directly after isolation, and either cultured alone or co-cultured with tumor organoids for 5 d (*n* = 3). Bar, 100 µm. (D) Representative electron microscopy image showing the proximity between organoids and PSCs in co-culture (*n* = 2). Bar, 5 µm. (E) H&E staining and Masson’s trichrome (MT) staining of fixed and paraffin-embedded organoids cultured alone or in co-culture with PSCs (*n* = 2). Bar, 50 µm. (F) Representative bright field and IF images of collagen I deposition (red) in organoid cultured alone or in co-culture with PSCs (*n* = 2). Bar, 200 µm. (G) Representative electron microscopy image of banded collagen fibrils (arrow), with fibril diameters ranging between 24 and 35 nm, in the extracellular space between organoids and PSCs (*n* = 2). Bar, 1 µm. (H) PSC proliferation curves plotting changes in mCherry intensity over time. Results show mean ± SD of two biological replicates. **, P < 0.01, unpaired Student’s *t* test. (I) Organoid proliferation curves plotting changes in GFP intensity over time. Results show mean ± SD of three biological replicates. *, P < 0.05, unpaired Student’s *t* test. (J) Passaging of organoids in different culture conditions in the presence or absence of PSCs. Complete media, DMEM/F12 supplemented with mitogens and growth factors. Reduced media, DMEM + 5% FBS. Red dot indicates the passage number when all organoids were found dead. Green dot indicates surviving organoids when the experiment was terminated. Each dot represents one biological replicate. Bars indicate the average number of passages for each condition. (K) RNA ISH of fixed and sectioned co-cultures for αSMA (*Acta2*, red) and *Krt18* (green) illustrating the spatial distribution of αSMA^high^ PSCs in comparison to Krt18^+^ (green) tumor organoids (*n* = 2). Counterstain, DAPI. Higher magnification on the right. Bars, 50 µm.

To investigate the interactions between cancer cells and PSCs, we established a three-dimensional organotypic co-culture system that combined GFP-labeled tumor-derived murine pancreatic organoids (Huch et al., 2013; Boj et al., 2015) and mCherry-labeled murine PSCs (Fig. 2 A). Pancreatic organoids are routinely cultured in a defined, mitogen-rich media. However, many factors that are present in this organoid media, including Noggin, B27 supplement, and TGBβ inhibitor, are known to be potent inhibitors of fibroblast proliferation. To avoid inhibition of PSC proliferation in co-cultures, we used a reduced media without these components, based on DMEM supplemented with 5% FBS. Although cancer-naive PSCs embedded alone in Matrigel remained quiescent, PSCs in co-culture with tumor organoids acquired a CAF phenotype, demonstrated by morphological activation with cellular extensions in close contact with tumor organoids (Fig. 2 B). Importantly, freshly isolated and nonimmortalized PSCs seeded directly in Matrigel showed similar morphological changes when co-cultured with organoids (Fig. 2 C), demonstrating that the activated phenotype in co-culture is an inherent feature of PSCs. Electron microscopy of co-cultures revealed the close relationship between cancer cells and PSCs, with only a thin gap filled with matrix separating the two cell types (Fig. 2 D). Activated PSCs deposited ECM (Fig. 2 E) containing collagen I (Fig. 2 F) that was organized into collagen fibrils (Fig. 2 G). Thus, our co-culture system is the first to recapitulate the desmoplastic reaction of PDA in vitro, with PSCs converting from a resting quiescent state to activated, stroma-producing CAFs.

PSCs were nonproliferative until organoids were included in co-culture (Fig. 2 H), and the co-cultures also promoted the proliferation of organoids (Fig. 2 I). Moreover, whereas organoid monocultures could be passaged indefinitely in complete media (Boj et al., 2015), reduced media conditions precluded prolonged passaging of organoids unless PSCs were present in co-culture (Fig. 2 J). The mutual proliferative benefits for both epithelial cells and PSCs are consistent with prior reports of similar symbiotic interactions that promote PDA (Bachem et al., 2005; Hwang et al., 2008; Vonlaufen et al., 2008; Sousa et al., 2016).

Analysis by ISH identified a clear distribution of αSMA^high^ PSCs surrounding the organoids in co-culture (Fig. 2 K), recapitulating the in vivo finding of a subpopulation of fibroblasts expressing high levels of αSMA (myCAFs) in close proximity to neoplastic cells (Fig. 1, A–D). This result also supports the premise that PSCs are a source of myofibroblastic CAFs in addition to BM-derived mesenchymal stem cells (BM-MSCs), which have previously been reported to give rise to myofibroblasts in cancer (Quante et al., 2011). Moreover, this novel co-culture platform confirms the existence of cooperative interactions between cancer cells and PSCs, and provides a way for their systematic characterization.

### A population of CAFs secretes inflammatory cytokines and loses myofibroblastic features

**Figure 3.**
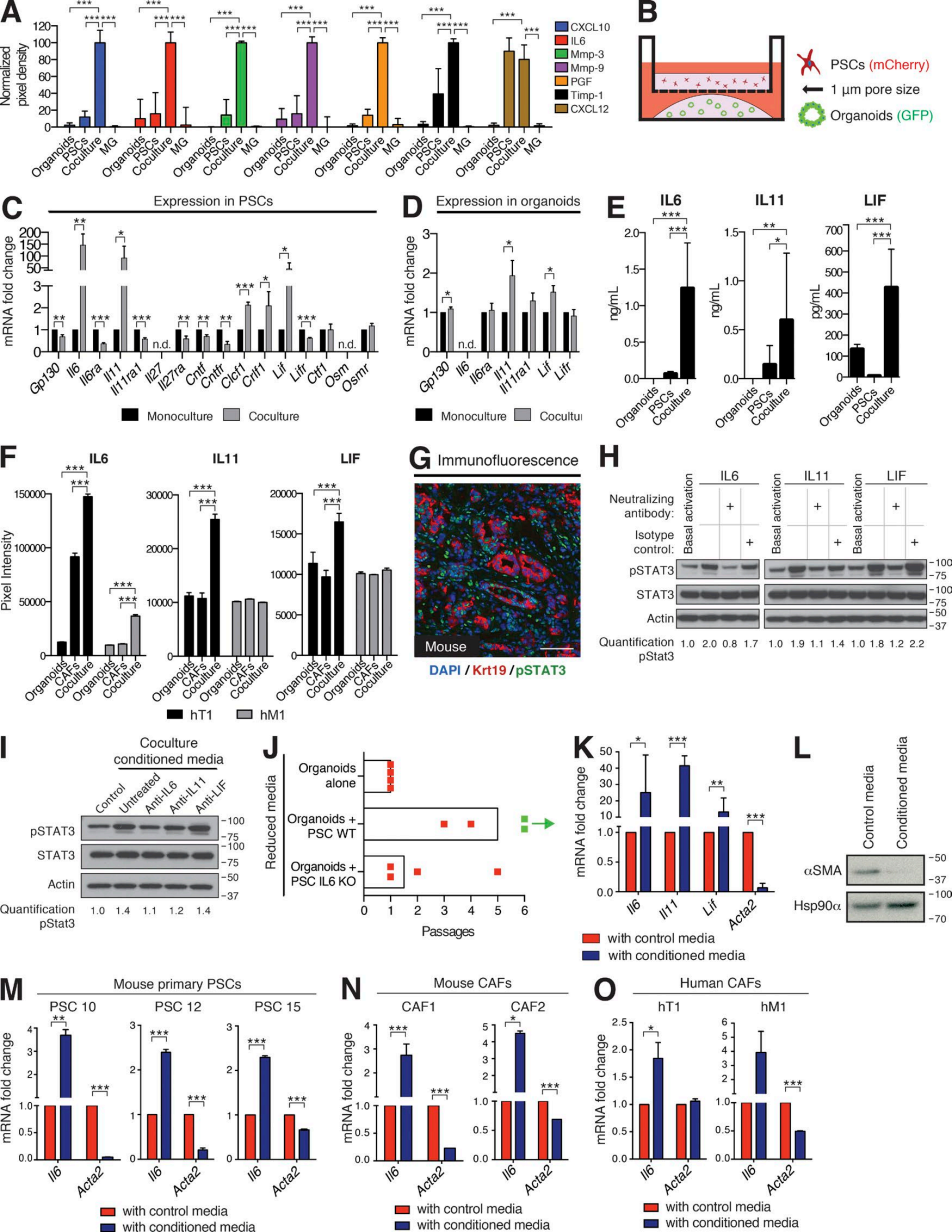
**Secretion of inflammatory cytokines from CAFs activates STAT3 in PDA organoids.** (A) Quantification of secretome dot blots of conditioned media from mouse tumor organoid monocultures, PSC monocultures, co-cultures, or Matrigel-only controls (MG). Results show normalized mean ± SD of three biological replicates. (B) Schematic illustration of the trans-well culture platform. (C and D) qPCR analysis of GP130 signaling ligands and receptors in mouse PSCs (C) or tumor organoids (D) cultured in monoculture or trans-well culture. Results show mean ± SD of four biological replicates. n.d., not detected. (E) ELISA of IL-6, IL-11, and LIF from conditioned media of PSC monocultures, organoid monocultures, or co-cultures. Results show mean ± SD of three biological replicates. (F) Quantification of secretome dot blots of conditioned media from human primary CAF monocultures, patient-matched tumor organoid monocultures, or co-cultures (*n* = 2). Results show mean ± SD of two technical replicates for each condition. (G) Representative IF image of KPC mouse tumor stained for phosphorylated STAT3 (Tyr705; green, pSTAT3) and the epithelial marker Cytokeratin 19 (Krt19, red; *n* = 2). Counterstain, DAPI (blue). Bar, 75 µm. (H) Western blot analysis of pSTAT3 in organoids treated with either 10 ng/ml recombinant IL-6, 10 ng/ml recombinant IL-11, or 50 ng/ml recombinant LIF, in the presence or absence of neutralizing antibodies or isotype controls (*n* = 2). Loading control, Actin. Molecular weights in kilodaltons. (I) Western blot analysis of pSTAT3 in organoids treated with co-culture conditioned media in the presence or absence of neutralizing antibodies against IL-6, IL-11, or LIF (*n* = 3). Loading control, Actin. Molecular weights in kilodaltons. (J) Passaging of organoids in reduced media conditions in monoculture or co-culture with WT (PSC WT) or IL-6 KO PSCs (PSC IL-6 KO). Red dot indicates the passage number when all organoids were found dead. Green dot indicates the passage number of surviving organoids when the experiment was terminated. Each dot represents one biological replicate. Bars indicate the average number of passages for each condition. (K) qPCR analysis of *Il6*, *Il11, Lif*, and *Acta2* transcript levels in PSCs cultured with control media (Matrigel-only conditioned media) or tumor organoid conditioned media. Results show mean ± SD of five biological replicates for *Il6, Lif*, and *Acta2*, and three biological replicates for *Il11*. (L) Western blot analysis of PSCs cultured with control media or tumor organoid conditioned media (*n* = 3). Loading control, Hsp90α. Molecular weights in kilodaltons. (M and N) qPCR analysis of *Il6* and *Acta2* in three primary PSC lines (M) and two KPC mouse CAFs (N) cultured with control media or tumor organoid conditioned media. Results show mean ± SD of two technical replicates for each line. (O) qPCR analysis for *IL6* and *ACTA2* transcript levels in human primary CAFs cultured with control media or conditioned media from the corresponding patient-matched tumor organoids. Results show mean ± SD of 2 technical replicates. *, P < 0.05; **, P < 0.01; ***, P < 0.001, unpaired Student’s *t* test.

To identify soluble factors that may mediate the symbiosis observed in co-culture, we analyzed the secretome of PSC and organoid co-cultures. We found that several secreted proteins, including inflammatory cytokines and chemokines, matrix remodeling proteins, and growth factors, were elevated specifically in co-culture (Fig. 3 A). One of the induced cytokines was interleukin 6 (IL-6), which has been reported to be up-regulated in PDA (Talar-Wojnarowska et al., 2009) and to promote cancer progression (Lesina et al., 2011; Zhang et al., 2013; Nagathihalli et al., 2016), cachexia, and immune suppression (Flint et al., 2016).

The increased cytokine production observed when tumor organoids and PSCs are co-cultured could be the result of a direct physical contact between these populations or alternatively, it could be caused by paracrine signaling between them. To disentangle these two possibilities and to determine which cell type was responsible for the increased cytokine production, we made use of a Transwell system, which allows paracrine interactions between tumor organoids and PSCs, but prevents direct contact between the two cell types (Fig. 3 B). IL-6 acts by binding to the IL-6 receptor subunit α (IL6RA), which in turn facilitates the dimerization and signaling of the GP130 signaling complex (Taga and Kishimoto, 1997). Because GP130 is known to interact with other ligand-receptor pairs, we measured the expression of additional GP130 signaling partners in both organoids and PSCs in trans-well cultures. Of the GP130 ligands found to be expressed in PSCs, *Il6*, *Il11*, and *leukemia inhibitory factor* (*Lif*) were the most highly up-regulated in trans-well cultures when compared with PSC monocultures (Fig. 3 C). Both IL-11 and LIF are reported to have roles in cancer progression (Putoczki et al., 2013; Marusyk et al., 2014), and LIF expression has been shown to be elevated in human PDA (Corcoran et al., 2011). In trans-well cultures, organoids did not express detectable levels of *Il6* but expressed *Il6ra*, *Il11ra1*, *Lifr*, and *Gp130* (Fig. 3 D), indicating that activated PSCs are the sole source of IL-6 in co-culture and that tumor organoids express the receptors needed to respond to PSC-secreted ligands. These results also confirm that direct contact with neoplastic cells is not required for PSCs to initiate cytokine secretion. Enzyme-linked immunosorbent assay (ELISA) was performed to confirm and quantify the elevated secretion of IL-6, IL-11, and LIF in co-culture (Fig. 3 E).

To evaluate these findings in human PDA fibroblasts, we isolated CAFs from primary (hT1) and metastatic (hM1) tumors of two PDA patients (Boj et al., 2015; Fig. S1, F–J). We then performed secretome analysis of conditioned media from co-cultures of human PDA CAFs with patient-matched tumor organoids. Secretion of IL-6 was induced in both primary tumor and metastatic co-cultures, whereas secretion of IL-11 and LIF was only induced in the co-culture derived from the primary tumor (hT1; Fig. 3 F).

Once dimerized with IL6RA, GP130 is phosphorylated and forms a complex with tyrosine kinases such as Janus kinases (JAKs). JAKs in turn phosphorylate and activate signal transducer and activator of transcription (STAT) factors, most notably STAT3, which plays key roles in cell growth and proliferation (Taga and Kishimoto, 1997). Immunofluorescence staining revealed detectable activation of STAT3 in KPC tumors, both in cancer cells and the surrounding stroma (Fig. 3 G). To confirm that tumor organoids activate STAT3 in response to paracrine stimuli, we measured STAT3 phosphorylation in organoids after the addition of recombinant IL-6, IL-11, and LIF to the media. All three ligands robustly activated STAT3 in organoids, and this effect was prevented when neutralizing antibodies against each ligand were added (Fig. 3 H). Interestingly, IL-6 was able to strongly activate STAT3 in tumor organoids, in contrast to studies using acinar cells isolated from *Kras^G12D^* mice that concluded that IL-6 trans-signaling is necessary to mediate robust STAT3 activation in pancreatic tumor cells (Lesina et al., 2011). STAT3 was also activated by the addition of co-culture conditioned media to tumor organoids in monoculture, and this activation was again blocked by addition of neutralizing antibodies, with the anti–IL-6 neutralizing antibody having the most prominent effect (Fig. 3 I).

As STAT3 activation is known to control cell survival and proliferation, we investigated its role in co-culture by using CRISPR/Cas9 gene editing to knockout IL-6 in two PSC lines. We confirmed loss of IL-6 secretion in PSCs by ELISA of conditioned media from trans-well cultures (Fig. S1 K). Interestingly, whereas organoids co-cultured with WT PSCs showed, as expected, prolonged passaging ability in reduced media conditions compared with monocultured organoids, continued passaging of organoids was impaired in co-cultures with IL-6–deficient PSCs (Fig. 3 J). These results demonstrate that tumor organoids in co-culture activate PSCs to secrete multiple factors, which in turn activate signaling pathways in organoids that sustain survival.

To further investigate the secretory phenotype of organoid-activated PSCs, we exposed quiescent PSCs to organoid-conditioned media (Fig. S2 A). In these conditions, PSCs acquired a CAF phenotype, indicated by morphological activation (Fig. S2 B), proliferation (Fig. S2 C), and up-regulation of *Il6*, *Il11*, and *Lif* mRNA levels (Fig. 3 K). Unexpectedly, we detected a simultaneous reduction in *Acta2* transcript and αSMA protein levels (Fig. 3, K and L). Although αSMA expression dropped in PSCs activated by tumor organoid-conditioned media, additional fibroblast surface markers, such as FAP and platelet-derived growth factor receptors (PDGFRs; Yuzawa et al., 2012) remained unchanged (Fig. S2, D and E), suggesting a specific loss of myofibroblastic features in cytokine-secreting PSCs. This result was reproduced in three mouse primary PSC lines (Fig. 3 M). Conditioned media from KPC cancer cells cultured in monolayer also induced this αSMA^low^ IL-6^high^ phenotype in PSCs, whereas conditioned media of NIH-3T3 fibroblasts did not (Fig. S2 F), suggesting that the ability to induce this secretory phenotype in PSCs is absent in cells of mesenchymal origin.

To extend our finding to mouse and human CAFs that already have been reprogrammed in vivo and not necessarily derived from PSCs, we isolated CAF lines from KPC mouse tumors, and validated their mesenchymal origin (Fig. S2, G–I). Mouse CAFs cultured with tumor organoid-conditioned media also increased *Il6* and concomitantly lost *Acta2* expression (Fig. 3 N). Although to a lesser extent, this pattern was also present in primary CAFs isolated from human primary and metastatic PDA samples when cultured with conditioned media from patient-matched tumor organoids (Fig. 3 O).

Previous studies have shown that pancreatic cancer cells induce IL-6 secretion in fibroblasts and PSCs (Erez et al., 2010; Feig et al., 2013; Zhang et al., 2013; Waghray et al., 2016), and that PSCs are a major source of IL-6 in PDA (Feig et al., 2013; Mace et al., 2016). Our data confirm this trait in cancer-naive PSCs that are allowed to interact with cancer cells, as well as in mouse and human PDA-derived CAFs. Importantly, for the first time we couple this phenotype with loss of myofibroblastic features. Such heterogeneity has previously been unappreciated, likely due to the fact that monolayers of fibroblasts uniformly express high levels of αSMA in culture.

Notably, the marked drop in bulk αSMA expression induced by organoid-conditioned media (Fig. 3 K) contrasted with the presence of CAFs expressing high levels of αSMA in proximity to neoplastic cells observed in co-cultures (Fig. 2 K) and in vivo (Fig. 1, A–D). We therefore hypothesized the coexistence of two distinct subtypes of CAFs in co-culture and PDA tissue, one being αSMA^high^ IL-6^low^ and proximal to the neoplastic cells (myCAFs), and the other being αSMA^low^ IL-6^high^ induced by paracrine signaling from the tumor compartment and more distantly distributed throughout the tumor. We termed these αSMA^low^ IL-6^high^ CAFs inflammatory CAFs (iCAFs) for their cytokine-secreting properties.

### CAFs coexist as two mutually exclusive and reversible subtypes

**Figure 4.**
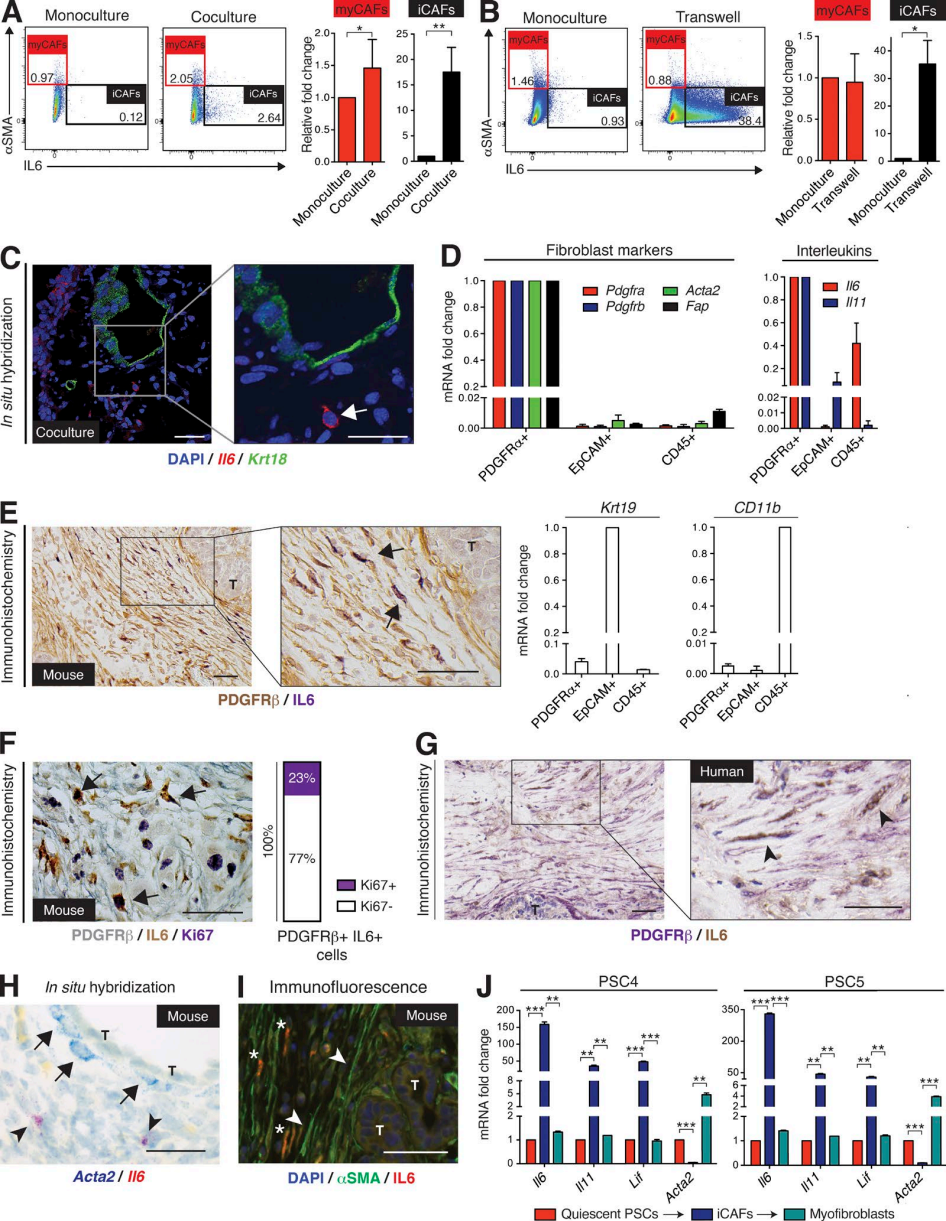
**Two mutually exclusive subpopulations of CAFs with reversible features coexist in pancreatic cancer.** (A and B) Flow cytometric analysis of αSMA and IL-6 in PSCs cultured alone or in either co-culture (A) or trans-well culture (B) with tumor organoids. Red frame indicates the gate defining myCAFs (αSMA^high^ IL-6^low^) and black frame indicates the gate defining iCAFs (αSMA^low^ IL-6^high^). Numbers indicate percentage of cells within marked gate. Graphs on the right are showing the fold change induction of myCAFs and iCAFs in co-culture, normalized to PSCs in monoculture. Results show mean ± SD of four (A) or two (B) biological replicates. *, P < 0.05; **, P < 0.01, unpaired Student’s *t* test. (C) Fluorescent RNA ISH of fixed and sectioned co-cultures for *Il6* (red) and *Krt18* (green), illustrating the spatial distribution of IL-6^+^ PSCs (iCAFs) with respect to KRT18^+^ tumor organoids (*n* = 2). Higher magnification on the right. Counterstain, DAPI (blue). Arrow indicates example of an iCAF. Bars, 50 µm. (D) qPCR analysis of interleukins (*Il6* and *Il11)* and markers of fibroblast (*Pdgfra, Pdgfrb, Acta2,* and *Fap*), epithelial (*Krt19*) and macrophage (*CD11b*) lineages in samples of primary cells sorted from KPC mouse tumors. Sorting was performed using three markers: PDGFRα (CD140a) for fibroblasts (*n* = 3), EpCAM for epithelial cells (*n* = 3) and CD45 for immune cells (*n* = 2). Results show mean ± SD of two to three biological replicates. All gene expression changes are statistically significant when compared with the reference population, P < 0.01, unpaired Student’s *t* test. (E) Representative image of sequential IHC for IL-6 (purple) and PDGFRβ (brown) in a KPC mouse tumor (*n* = 3). Arrows indicate double positive cells. T, tumor gland. Bars, 50 µm. (F, left) Representative image of sequential IHC for PDGFRβ (gray), IL-6 (brown), and Ki67 (purple) in a KPC mouse tumor (*n* = 3). Arrows indicate examples of triple positive cells. Bar, 50 µm. (right) Quantification of Ki67 staining in PDGFRβ^+^/IL-6^+^ cells (iCAFs), total of 593 cells were counted. (G) Representative image of sequential IHC for IL-6 (brown) and PDGFRβ (purple) in a human PDA (*n* = 6). Arrowheads indicate double positive cells. T, tumor gland. Bars, 50 µm. (H) Representative image of RNA ISH for *Acta2* (blue) and *Il6* (red) in KPC mouse tumors (*n* = 4). Arrows indicate examples of *Acta2*-positive cells in the periglandular area, arrowheads indicate examples of *Il6*-positive cells further away from neoplastic cells. Bar, 50 µm. T, tumor glands. (I) Representative IF image for αSMA (green) and IL-6 (red) in a KPC mouse tumor (*n* = 3). Counterstain, DAPI (blue). Arrowheads indicate examples of αSMA-positive cells in the periglandular area; * indicates examples of IL-6–positive cells further away from neoplastic cells. Bar, 50 µm. T, tumor glands. (J) qPCR analysis of *Il6*, *Il11*, *Lif*, and *Acta2* transcript levels in two PSC lines (PSC4 and PSC5) first grown as monocultures in Matrigel (quiescent PSCs), then transferred to trans-well cultures with tumor organoids (iCAFs), and finally plated as monolayer cultures (myofibroblasts). Results show mean ± SD of two technical replicates for each PSC line. **, P < 0.01; ***, P < 0.001, unpaired Student’s *t* test.

To determine whether distinct CAF subtypes coexist in co-culture, flow cytometry was used to detect αSMA and IL-6 expression in PSCs co-cultured with tumor organoids (Fig. S2 J). As our hypothesis predicted, co-culture triggered high αSMA expression only in a subset of PSCs (myCAFs), whereas a separate population showed low αSMA expression and concomitantly elevated IL-6 expression (iCAFs; Fig. 4 A). Moreover, we found that trans-well cultures of PSCs and organoids (Fig. 3 B) markedly induced iCAF formation, but did not cause an increase in myCAFs (Fig. 4 B). This demonstrates that the formation of myCAFs is contact-dependent, whereas iCAFs may be enriched in the absence of contact with PDA cells. The presence of iCAFs in co-culture was evident by ISH of *Il6* (Fig. 4 C), which also confirmed that these CAFs were spatially separated from organoids.

To evaluate whether CAFs produce interleukins in vivo, we dissociated KPC mouse tumors into single cells, isolated CAFs by flow cytometry, and characterized their gene expression. We sorted CAFs based on PDGFRα expression because it has been shown to be a specific surface marker for CAFs (Erez et al., 2010). As a comparison, epithelial and immune cells were isolated, using the EpCAM and CD45 surface markers, respectively (Fig. S2 K). No overlap was detected between the CD45^+^ population and the PDGFRα^+^ population (Fig. S2 L), demonstrating the mutual exclusivity of these two markers. We further confirmed the purity of the three sorted cell populations by qPCR for additional fibroblast, epithelial and immune markers, and demonstrated that PDGFRα**^+^** CAFs contain the majority of IL-6 transcripts in PDA (Fig. 4 D). Additionally, we found that IL-11 mRNA is also predominantly present in CAFs, further supporting the presence of an inflammatory CAF phenotype (Fig. 4 D).

To investigate the spatial distribution of IL-6^high^ CAFs in vivo, immunohistochemical analysis of KPC tumor tissue was performed. Consistent with the co-cultures, we detected IL-6 expression in cells that were located further away from neoplastic cells in the desmoplastic stroma (Fig. 4 E). We confirmed that these were iCAFs by co-staining for PDGFRβ, another fibroblast marker (Yuzawa et al., 2012; Fig. 4 E). Importantly, a fraction of these iCAFs proliferate in vivo, as indicated by Ki67 coexpression (Fig. 4 F), demonstrating the lack of a senescence-associated secretory phenotype (Coppé et al., 2010). Furthermore, we could identify IL-6–expressing CAFs in human PDA by co-staining human tumor tissues for IL-6 and PDGFRβ, confirming that iCAFs are also an inherent trait of human PDA (Fig. 4 G). To confirm the spatial distribution of myCAFs and iCAFs in KPC tumors, we used ISH and immunofluorescence staining, and demonstrated that periglandular αSMA^high^ cells were spatially separated from the more distant IL-6^high^ cells (Fig. 4, H and I), further supporting our observation of two distinct CAF populations in vivo.

To determine if the iCAF phenotype was permanent or transient, we plated myofibroblastic PSCs from monolayers into Matrigel to obtain quiescent PSCs, and then induced the iCAF phenotype by culturing these cells in trans-well with PDA organoids. As PSCs grown in monolayers are known to obtain myofibroblastic features, we subsequently plated the iCAFs in monolayer, and found that iCAFs rapidly reverted to a myofibroblastic state, down-regulating *Il6*, *Il11*, and *Lif*, and simultaneously up-regulating *Acta2* expression levels (Fig. 4 J). This observation suggests that CAFs are dynamic and can assume different phenotypes based on their spatial and biochemical niche within the PDA microenvironment.

Overall, our results, both in vivo and in co-culture, show a spatial separation between iCAFs and myCAFs. MyCAFs are located in the periglandular region, suggesting that direct juxtacrine interactions with cancer cells are required for myCAF formation. iCAFs, on the other hand, are induced by secreted factors from cancer cells and are located more distantly from neoplastic cells and myCAFs in PDA. Although we have demonstrated the existence and spatial separation of these two CAF populations, other CAF populations with unique genetic signatures may exist. The existence of these diverse populations and their functions in tumors remain to be elucidated.

### Inflammatory CAFs and myofibroblasts show distinct transcriptional profiles

**Figure 5.**
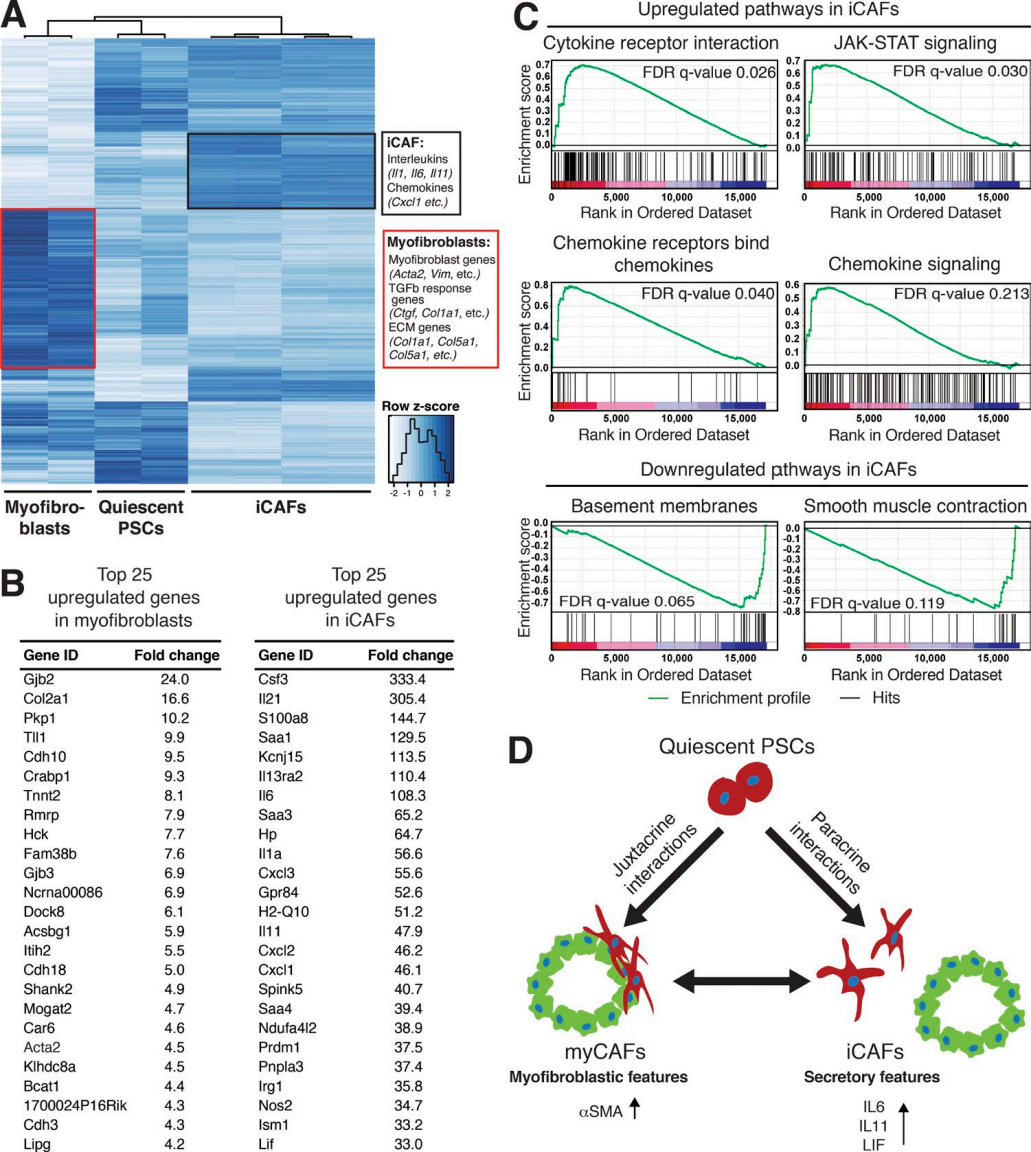
**Inflammatory CAFs and myofibroblasts have distinct transcriptional profiles.** (A) RNA sequencing analysis of quiescent PSCs (PSCs embedded alone in Matrigel; *n* = 2), iCAFs (PSCs grown in trans-well culture with tumor organoids; *n* = 4) and myofibroblasts (PSCs grown in monolayer; *n* = 2). The heat map shows differentially expressed genes between the three cell states. Uniquely expressed genes for iCAFs and myofibroblasts are indicated in the boxes. Adjusted P < 0.01. (B) Lists of the 25 most up-regulated genes in iCAFs and myofibroblasts compared with quiescent PSCs. Adjusted P < 0.05. (C) GSEA of most up-regulated and down-regulated pathways in iCAFs compared with quiescent PSCs. (D) Working model illustrating the dynamic relationship between quiescent PSCs, myCAFs and iCAFs.

To better understand the differences between these subtypes, we compared the transcriptomes of quiescent PSCs (PSCs embedded alone in Matrigel), iCAFs (SMA^low^ IL-6^high^ PSCs cultured in trans-well with tumor organoids), and myofibroblastic PSCs (SMA^high^ IL-6^low^ PSCs grown in monolayer) as a proxy for myCAFs, because obtaining myCAFs from tumors or co-cultures presents a technical challenge (Fig. S2 M). By RNA sequencing, we found clusters of genes uniquely up-regulated in either myofibroblasts or iCAFs (Fig. 5, A and B; Fig. S2 N; and Table S1). In particular, *Acta2* and TGFβ response genes, such as *Ctgf* and *Col1a1*, were up-regulated in myofibroblasts compared with quiescent PSCs and iCAFs. On the other hand, cytokines, such as *Il6*, *Il11*, and *Lif*, and chemokines, such as *Cxcl1* and *Cxcl2*, were uniquely up-regulated in iCAFs (Fig. 5, A and B; and Table S1). Furthermore, Gene Set Enrichment Analysis (GSEA) of iCAFs compared with quiescent PSCs confirmed the up-regulation of cytokine signaling pathways, and identified JAK/STAT signaling as one of the most significantly up-regulated pathways in iCAFs (Fig. 5 C). Additionally, as expected, basement membranes and smooth muscle contraction pathways were the most significantly down-regulated ones in iCAFs (Fig. 5 C). The identification of unique transcriptional signatures of myofibroblasts and iCAFs further supports the model that CAFs within the PDA microenvironment acquire distinct phenotypes. In particular, the transcriptomic profiling implies that myofibroblasts are contractile and stroma remodeling, whereas iCAFs are characterized by a secretory phenotype, with the ability to modulate in a paracrine manner cancer cells and other cell types present in the tumor. Importantly, secreted factors from iCAFs, such as IL-6, likely contribute to systemic effects in PDA patients, such as cachexia and immune suppression (Feig et al., 2013; Flint et al., 2016; Mace et al., 2016).

In summary, we have identified two spatially separated, reversible, and mutually exclusive subtypes of CAFs (Fig. 5 D). iCAFs, which are activated by paracrine factors secreted from cancer cells, are located more distantly from neoplastic cells within the dense tumor stroma. Although iCAFs still retain expression of αSMA, they are characterized by significantly lower αSMA levels compared with myCAFs, and instead intensely elevate expression of cytokines and chemokines with known roles in cancer progression and disease pathophysiology. Indeed, iCAFs are a significant source of IL-6 and IL-11 in the PDA microenvironment (Fig. 4 D), with the ability to stimulate the STAT3 pathway in cancer cells (Fig. 3 I). However, myCAFs are defined by high αSMA expression and periglandular location (Fig. 1, A–D), and their formation appears dependent on juxtacrine interactions with cancer cells (Fig. 4 B). Furthermore, myCAFs lack the expression of inflammatory cytokines, distinguishing them from iCAFs. Importantly, both inflammatory and myofibroblastic CAFs can be generated from PSCs, and can dynamically reverse from one cell state to the other (Fig. 4 J). The detailed mechanisms that govern the formation and transition of these cell states will require further investigation.

Although we have identified two subtypes of PSC-derived CAFs in PDA, it is likely that additional CAF subtypes with distinct roles in the pathophysiology of this disease exist. Indeed, our data already reveal a large population of CAFs with low expression of both αSMA and IL-6 (Fig. 4, A and B). Furthermore, a more thorough definition of stromal subtypes may involve additional factors and more complex genetic signatures. Importantly, although other groups have identified different subtypes of stroma in PDA across patients (Moffitt et al., 2015), our work is the first to characterize the intratumoral CAF heterogeneity in PDA.

The concept of intratumoral CAF heterogeneity may address the conflicting reports that have emerged in the field in regard to CAF functions. Indeed, in recent years, different approaches to target the stroma have given contradicting results and, at times, promoted worse outcomes (Lee et al., 2014; Özdemir et al., 2014; Rhim et al., 2014). For instance, attempts to deplete CAFs based on their αSMA expression have led to decreased survival in tumor-bearing mice (Özdemir et al., 2014). However, given our results, this approach may have preferentially eliminated myCAFs, while leaving other CAF populations intact. Therefore, the traditional view of the tumor stroma as a uniformly protumorigenic niche calls for reconsideration, as certain CAF subtypes might have protumorigenic properties, whereas others might have antitumorigenic features. Therapeutic development must consider this possibility to provide optimal benefits to PDA patients.

## Materials and methods

### PSC isolation

PSCs were isolated from WT C57BL/6J mice as previously described (Apte et al., 1998) with minor modifications. In brief, pancreata were minced and digested for 30 min at 37°C in a dissociation buffer containing 0.05% collagenase P (Sigma-Aldrich) and 0.1% DNase I (Sigma-Aldrich) in Grey’s balanced salt solution (GBSS; Sigma-Aldrich). Digested pancreata were filtered through a 100-µm nylon mesh and washed in GBSS with 0.3% BSA and 0.1% DNase I. After spinning, the cell pellet was resuspended in 9.5 ml GBSS with 0.3% BSA and 43.75% Histodenz (Sigma-Aldrich). 6 ml of GBSS with 0.3% BSA was layered on top of the cell suspension, and the gradient was centrifuged for 20 min at 1,400 RCF (with break switched off). The cells in the fuzzy band just above the interface between the Histodenz and GBSS were harvested, washed in PBS, and plated.

### Mouse models

KPC mice (*Kras^LSL-G12D/+^; Trp53^LSL-R172H/+^; Pdx-1-Cre*) have previously been described (Hingorani et al., 2005). Each of the three alleles in the KPC mouse strain (Kras; Trp53 and Pdx1-Cre) were backcrossed individually onto the C57BL/6J mouse strain obtained from The Jackson Laboratory (stock number 000664) for at least 20 generations. To generate the KPC mouse model, mice carrying single alleles were crossed onto each other. The Rosa26-LSL-YFP allele (Srinivas et al., 2001) was backcrossed onto the C57BL/6J mouse strain obtained from The Jackson Laboratory and introduced into the KPC C57BL/6J strain for a total of at least 20 generations to generate KPC; *Rosa26^LSL-YFP/+^* (KPCY). NOD *scid* gamma (NSG) mice were purchased from The Jackson Laboratory (stock number 005557). All animal procedures and studies were conducted in accordance with the Institutional Animal Care and Use Committee (IACUC) at CSHL.

### Mouse organoid isolation and culture

Mouse tumor organoids were isolated from KPC mice with histologically verified PDA. Tumor tissue was minced and digested at 37°C for 12 h in a dissociation buffer containing 0.012% (wt/vol) collagenase XI (Sigma-Aldrich) and 0.012% (wt/vol) dispase (Gibco) in DMEM (Gibco) containing 1% FBS (Gibco). The tissue debris was allowed to settle, and the dissociated cells were pelleted and washed in Advanced DMEM/F12 (Invitrogen) and seeded in growth factor-reduced Matrigel (BD). Organoids were cultured in complete organoid media (Boj et al., 2015); Advanced DMEM/F12 supplemented with 1x GlutaMAX (Gibco), 1x Hepes (Gibco), 1x B27 (Invitrogen), 1.25 mM N-Acetylcysteine (Sigma-Aldrich), 10 nM gastrin (Sigma-Aldrich), 50 ng/ml EGF (PeproTech), 10% RSPO1-conditioned media, 100 ng/ml Noggin (PeproTech), 100 ng/ml FGF10 (PeproTech), and 10 mM Nicotinamide (Sigma-Aldrich).

To passage, organoids were washed out from the Matrigel using cold Advanced DMEM/F12 supplemented with 1x GlutaMAX (Gibco) and 1x Hepes (Gibco), and mechanically dissociated into small fragments using fire-polished glass pipettes, and then seeded into fresh Matrigel. Passaging was performed at a 1:8 split ratio roughly twice per week. To create frozen stocks, organoids were passaged and mixed with Recovery Cell Culture Freezing Medium (Gibco) and cryopreserved using standard procedures. Cultures were thawed using standard thawing procedures, washed once with Advanced DMEM/F12 supplemented with 1x GlutaMAX (Gibco) and 1x HEPES (Gibco), and seeded in Matrigel with organoid media supplemented with 10.5 µM Y-27632 (Sigma-Aldrich) for the first passage.

### Mouse CAF isolation

Mouse CAFs were isolated from KPC mice with histologically verified PDA. CAFs were isolated from tumors using a combination of outgrowth and clonal isolation. The edge of the tumor mass was minced and dissociated in DMEM containing 1% FBS, 0.125 mg/ml collagenase (Sigma-Aldrich), and 0.125 mg/ml dispase (Life Technologies) for 1.5 h at 37°C in a thermomixer. The sample was trypsinized for 10 min then quenched in 10% FBS/DMEM. The pellet containing tumor pieces was plated on a 6-cm dish, and fibroblasts were allowed to grow out and attach to the plastic. To avoid cancer cell contamination, after the cell culture was established and passaged at least 10 times, cells were plated in high dilution in a 96-well plate to obtain one cell per well, and single clones were expanded. Validation of fibroblast identity was performed at the protein level by flow cytometry analysis for fibroblast surface markers, and at the genomic level by PCR to verify presence of WT and mutant *Kras* alleles. The primers used are: 5': GGGTAGGTGTTGGGATAGCTG, and 3': TCCGAATTCAGTGACTACAGATGTACAGAG, giving a 285-bp band for WT and 325-bp band for mutant Kras.

### Human organoid and CAF isolation

All human organoid experiments were approved by the IRBs of MSKCC, MDACC, and CSHL, and all subjects taking part in the study provided written informed consent. Tumor tissue was minced and digested in a rotating shaker with collagenase II (5 mg/ml; Gibco) for 12 h at 37°C in human complete media, i.e., complete media (Boj et al., 2015), supplemented with 50% Wnt3a-conditioned media and 1 µM Prostaglandin E2 (Tocris; Boj et al., 2015). The tissue was further digested for 15 min at 37°C in TrypLE (Gibco) in a rotating shaker. The digested tissue was washed repeatedly after digestion. 10% of the tissue suspension, including larger undigested pieces, was plated in 10% FBS/RPMI on 10-cm dishes to allow fibroblast outgrowth. The remaining tissue suspension was seeded in Matrigel and cultured in human complete medium for establishment of organoids. Fibroblast outgrowth was observed in the 10-cm dishes within 48 h. Fibroblast identity was confirmed at the DNA level by Sanger sequencing to verify the WT status of *KRAS* at codon 12 (forward primer; 5′-CTGGTGGAGTATTTGATAGTG-3′, reverse primer; 5′-CTGTATCAAAGAATGGTCCTG-3′). To confirm that the established CAF lines had not become transformed in the isolation process, CAFs (2.0–2.5 × 10^5^ cells) were resuspended in 100 µl Matrigel and injected subcutaneously in flanks of 6–8-wk-old NOD SCID gamma mice (The Jackson Laboratory).

### Mouse KPC tumor cell isolation for monolayer cultures

The primary tumor cells used in monolayer experiments were isolated from KPC tumors by an outgrowth method. In brief, 30–50 mg pieces of tumors were minced in DMEM. The minced tissue was then transferred into 5 ml of DMEM with 2 mg/ml Collagenase V (Sigma-Aldrich) and incubated for 45 min at 37°C in a thermomixer, with vortexing every 5–10 min. After digestion, 10 ml of 10% FBS/DMEM was added, and samples were pelleted, resuspended in 10% FBS/DMEM, and plated on a 10 cm dish. Cultures were passaged at a high split ratio (1:80) for several passages to favor for the growth of neoplastic cells.

### Cell culture conditions for monolayer cultures

KPC mouse CAFs, mouse PSCs, and KPC primary tumor cells were cultured in DMEM containing 5% FBS, 1% l-glutamine, and 1% Penicillin/Streptomycin. NIH-3T3 fibroblasts were cultured in DMEM containing 10% FBS, 1% l-glutamine, and 1% Penicillin/Streptomycin. Human CAFs were cultured in RPMI 1640 (Gibco) containing 10% FBS, 1% l-glutamine, and 1% Penicillin/Streptomycin. All cells were cultured at 37°C with 5% CO_2_.

### Transfection of fluorophores and immortalization

To immortalize primary PSCs and human CAFs, the SV40 large T-antigen was cloned from pBABE-puro SV40 LT (Addgene) into pLVX-IRES-tdTomato (Takara Bio Inc.) using the BamHI site. 293T cells were transfected with the pLVX-SV40 LT-IRES-tdTomato plasmid together with psPAX2 and pMD2.G packaging plasmids using X-treme GENE 9 DNA Transfection Reagent (Roche). Media was changed 24 h later, and lentivirus supernatant was collected after an additional 24 h. The supernatant was filtered through a 0.45-µm filter and aliquots were kept at –80°C. Primary PSCs and human CAFs were grown to 70% confluency, and infected with the virus supernatant for 24 h. Virus was aspirated and fresh media was added. After passaging, tdTomato was visualized by fluorescent microscopy to verify infection efficiency.

To obtain GFP-labeled organoids, PGK-Neo-IRES-EGFP retrovirus was produced in ecotropic Phoenix cells, concentrated with RetroX concentrator (Takara Bio Inc.), and resuspended in Advanced DMEM/F12 supplemented with 10 µM Y-27632 (Sigma-Aldrich). 5 × 10^4^ cells were resuspended in concentrated retrovirus and spinoculated at 600 RCF for 1 h at room temperature, washed, and then seeded into Matrigel. To obtain mCherry-labeled PSCs or PGK-Neo-IRES-mCherry, retrovirus was produced in ecotropic Phoenix cells and incubated with PSCs grown in monolayer for 24 h. 2 d after infection, cells were treated with 1 mg/ml G418 (Gibco) for selection.

### IL-6 CRISPR/Cas9-mediated knockout

To knock out IL-6, lenti-Cas9-Blast plasmids (Addgene Plasmid #52962) were transfected into 293T cells to produce lentivirus. The virus was concentrated using Lenti-X Concentrator (Takara Bio Inc.), and resuspended in DMEM with 5% FBS. Immortalized PSCs were infected with the resuspended virus and selected using 2 µg/ml blasticidin (Thermo Fisher Scientific) to create PSC lines stably expressing Cas9. Short guide RNAs (sgRNAs) against IL-6 (5′-CACCTATACCACTTCACAAGTCGG-3′ and 5′-CACCTAAGCCTCCGACTTGTGAAG-3′) were designed using the CRISPR GRNA Design Tool (Atum) and cloned into the LRG plasmid (Lenti-sgRNA-EFS-GFP; Addgene; Shi et al., 2015). Next, LRG lentivirus was produced in 293T cells, concentrated using Lenti-X Concentrator (Takara Bio Inc.), and resuspended in DMEM with 5% FBS. Cas9-expressing PSCs were infected with resuspended LRG virus and efficient cleavage by the sgRNAs was confirmed using the SURVEYOR assay (Transgenomic), following the manufacturer's protocol using a PCR primer set specific to the sgRNA targeted region of IL-6 (forward primer; 5′-CCTCTGGCGGAGCTATTGAG-3′, reverse primer; 5′-CCAGACAGGAAAGGAACCCC-3′). PSCs were then plated as single clones in 96-well plates, and the clones were allowed to expand. Clones that were found to be GFP positive were then analyzed for loss of IL-6. Deletion of IL-6 was confirmed by Sanger sequencing, and by IL-6 ELISA (R&D Systems) of conditioned media from PSCs grown in trans-well cultures with mouse tumor organoids. IL-6–negative clones were combined into IL-6 KO lines, and clones positive for IL-6 secretion were combined into control lines. In total, two IL-6 KO lines and two control lines were made from two parental PSC lines.

### Co-culture and passaging of tumor organoids and CAFs

For co-cultures, mouse or human organoids were split at a 1:6 ratio and mixed with 1x10^4^ PSCs or CAFs, seeded in Matrigel, and cultured in DMEM containing 5% FBS and 1% Penicillin/Streptomycin. For passaging experiments, established co-cultures and monocultures were split every 7 d at a 1:4 ratio and fresh medium was added. For trans-well cultures, 2 × 10^4^ PSCs or human CAFs were seeded in Matrigel on top of the trans-well membrane (1 µm pore size, Greiner Bio-One) with organoids growing in the lower compartment in 24-well plates.

### Conditioned media experiments

For conditioned media experiments, organoids or monolayer cells were cultured for 3–4 d in DMEM containing 5% FBS, 1% l-glutamine, and 1% Penicillin/Streptomycin. Media was collected, spun down to remove debris, and added to PSCs plated in Matrigel.

### Flow cytometry and cell sorting

For flow cytometric analysis of mouse CAF and PSC lines, cells were grown in DMEM containing 5% FBS, trypsinized, and subjected to surface staining with anti-FAP (R&D Systems; 1:40 for PSCs; Abcam; 1:50 for mouse CAFs) or anti–mouse CD326 (EpCAM) APC (eBioscience clone G8.8; 1:160). Subsequent antibodies used for FAP staining are anti–goat/sheep IgG–biotin (Sigma-Aldrich; 1:50) and streptavidin APC-Cy7 (BD; 1:200), Goat anti–rabbit Alexa Fluor 488 and goat anti–rabbit Alexa Fluor 568 (Thermo Fisher Scientific; 1:500). Human CAFs were grown in RPMI containing 10% FBS, and stained and analyzed similarly, using the FAP antibody obtained from R&D Systems.

For intracellular flow cytometric staining, 5 × 10^5^ cells were treated with GolgiPlug (BD) for 6 h before fixation and permeabilization with Cytofix/Cytoperm kit (BD) according to the manufacturer’s instructions. Cells were stained with the appropriate antibodies in FACS buffer (PBS containing 0.2% BSA, 2 mM EDTA, and 0.02% sodium azide) at 4°C for 30 min in the dark. The following conjugated antibodies were used: APC-Cy7-anti-αSMA (1A4; Abcore) and PE-anti–IL-6 (MP5-20F3; BioLegend). Fixable viability dye eFluor450 (eBioscience) was used to differentiate between dead and live cells. The stained populations were analyzed using an LSR-II flow cytometer (BD) and FlowJo software (Tree Star; Version 10). At least 20,000 events were recorded per condition.

For sorting of CAFs from KPC tumors, tumors (>8 × 8 mm in diam) were minced and digested in DMEM containing 10% FBS, 2.5 mg/ml Collagenase D (Roche), 0.5 mg/ml Liberase (Roche), and 0.2 mg/ml DNase I (Sigma-Aldrich), for 45 min at 37°C in a rotating shaker. Tumor pieces were then strained through a 40-µm cell strainer, and red blood cells were lysed using ACK lysis buffer for 4 min on ice. After neutralization with flow buffer (5% FBS/PBS), cells were blocked with rat anti–mouse CD16/CD32 (Fc Block; BD; 1:100 for 20 min), and then subjected to staining with anti–mouse CD45-BV510 (BioLegend; clone 30-F11; 1:100), anti–mouse CD326 (Ep-CAM)-Alexa Fluor 488 (BioLegend; clone G8.8; 1:100), anti-mouse CD31-PE/Vio770 (Miltenyi Biotec; clone 390; 1:10), and anti–mouse CD140a (PDGFRα)-APC (BioLegend; clone APA5; 1:100) for 30 min on ice. Cells were sorted on the FACSAria cell sorter (BD) for CD45^+^, EpCAM^+^, and PDGFRα^+^ populations.

### qPCR

1 µg RNA was used to reverse transcribe cDNA using the TaqMan reverse transcription reagents (Thermo Fisher Scientific). qPCR was performed using gene-specific TaqMan probes (Applied Biosystems) and master mix (Thermo Fisher Scientific), following the manufacturer’s instructions. Gene expression was normalized to Hprt. The following TaqMan Probes were used (Mm, mouse probes; Hs, Human probes): Acta2, Mm01546133_m1 and Hs00426835_g1; Cd11b, Mm00434455_m1; Clcf1, Mm01236492_m1; Cntf, Mm04213924_s1; Cntfr, Mm00516693_m1; Col1a1, Mm00801666_g1; Crlf1, Mm00517026_m1; Ctf1, Mm00432772_m1; Ctgf, Mm01192932_g1; Fap, Mm01329177_m1; Gp130, Mm00439665_m1; Has2, Mm00515089_m1; Hprt, Mm00446968_m1, Hs02800695_m1; Il11, Mm00434162_m1; Il11ra1, Mm01218402_m1; Il6, Mm00446190_m1, Hs00985639_m1; Il6ra, Mm00439653_m1; Il27, Mm00461162_m1; Il27ra, Mm00497259_m1; Krt19, Mm00492980_m1; Lif, Mm00434761_m1; Lifr, Mm00442942_m1; Mmp2, Mm00439498_m1; Osm, Mm01193966_m1; Osmr, Mm01307326_m1; Pdgfra, Mm00440701_m1; Pdgfrb, Mm00435546_m1; Vdr, Mm00437297_m1.

### Oil Red-O staining

Immortalized PSCs were cultured on plastic or embedded in Matrigel for 4 d and fixed in 10% formalin for 10 min at room temperature, followed by 5-min incubation in 60% isopropanol. After drying, the cells were incubated with filtered Oil Red-O working solution for 10 min. The working solution was prepared by dissolving 0.35 g Oil Red-O (Sigma-Aldrich) in 100 ml isopropanol, which was then diluted in water at a 3:2 ratio. After staining, the cells were washed four times with water and imaged.

### TGFβ stimulation

Cells were treated with 20 ng/ml of recombinant human TGFβ1 (Sigma-Aldrich) for 4–5 d before RNA isolation and qPCR analysis.

### Immunofluorescence staining of monocultures and co-cultures

Cultures were fixed with 2% PFA for 20 min at room temperature, and washed three times for 10 min in 1x PBS/Glycine solution (10× stock: 38.0 g NaCl, 9.38 g Na_2_HPO_4_, 2.07 g NaH_2_PO_4_, and 37.5 g glycine, in 500 ml PBS). Three washes in 1× wash solution were performed (10× stock: 38.0 g NaCl, 9.38 g Na_2_HPO_4_, 2.07 g NaH_2_PO_4_, 2.5 g NaN_3_, 5.0 g BSA, 10 ml Triton X-100, and 2.5 ml Tween-20, in 500 ml PBS), followed by 1 h of blocking in 1× wash solution with 10% horse serum. The cultures were then incubated over night at 4°C with rabbit anti-collagen I antibody (ab34710, Abcam) diluted 1:200 in 1x blocking solution. After three washes, the cells were incubated for 1 h with Alexa-568 Goat anti-Rabbit IgG antibody (Invitrogen) diluted 1:1000 in 1× blocking solution. Counterstain, DAPI (Sigma-Aldrich). After three washes, the slides were mounted and imaged.

### ISH

RNA ISH was performed on freshly prepared formalin-fixed paraffin-embedded (FFPE) tissue sections (6 µm thickness) using the ViewRNA ISH Tissue 2-Plex Assay (Affymetrix), according to the manufacturer's instructions using 10-min pretreatment and protease treatment incubation times. ISH probes used were as follows: mouse αSMA, VB1-16010-01; human αSMA, VA1-10300; mouse FAP, VB1-16010-01; mouse IL-6, VB1-10012; mouse Krt18, VB6-11059; human Krt18, VA6-11561.

### Immunohistochemical/immunofluorescent staining of tissues

All stainings were performed on 5-µm sections of mouse and human tissues. Hematoxylin and Eosin (H&E) staining was performed according to standard protocols. For immunohistochemistry (IHC), sections were deparaffinized, and antigen retrieval was performed in a pressure cooker in 10 mM sodium citrate buffer, pH 6.0. 3% H_2_O_2_ was used to block endogenous peroxidases. Primary antibodies used for IHC were as follows: IL-6 (1:50 for mouse; Cell Signaling Technology; 1:100 for human; Abcam), GFP (to detect YFP; 1:1,000; Abcam), Ki67 (1:250; Thermo Fisher Scientific), PDGFRβ (1:400 for mouse and 1:200 for human; Abcam), and αSMA (1:200; Abcam). Hematoxylin was used as nuclear counterstain. For double and triple IHC on mouse tissue, a sequential IHC protocol was applied (Vector Laboratories) and the substrates used were as follows: ImmPACT DAB peroxidase (brown), ImmPACT VIP peroxidase (purple), and ImmPACT SG peroxidase (gray). The same protocol was used for double IHC staining of IL-6 and PDGFRβ on human tissue, using an automated Ventana Benchmark staining machine (Ventana Medical Systems).

For IF of pSTAT3 in KPC tumors, frozen sections were first fixed in 3% formaldehyde for 15 min at room temperature, and then fixed in methanol for 10 min at –20°C. Slides were immediately washed in PBST (1% Tween-20, PBS) after fixation and blocked with 10% goat serum in PBST and 0.3% Triton X-100 for 30 min at room temperature. Slides were then stained with Phospho-Stat3 (1:200; Cell Signaling Technology) and Krt19 (1:200; DSHB TROMA-III) antibodies for 1 h at room temperature. Slides were washed in PBST, and then stained with anti–rabbit Alexa Fluor 488 (1:500; Thermo Fisher Scientific), and anti–rat Alexa Fluor 568 (1:500; Thermo Fisher Scientific) secondary antibodies for 45 min at room temperature. After washing in PBST, sections were stained with DAPI for 5 min at room temperature and coverslipped with Histomount Mounting Solution (Thermo Fisher Scientific).

For IF of αSMA and IL-6, FFPE slides were used, and deparaffinization and antigen retrieval were performed according to the IHC protocol. Slides were incubated with primary antibodies for αSMA (1:100; Dako) and IL-6 (1:50; Cell Signaling Technology) overnight at 4°C. Slides were washed with TBS (Tris-buffered saline) and stained with anti–mouse Alexa Fluor 488 and anti–rabbit Alexa Fluor 568 (1:1,000; Invitrogen) secondary antibodies for 1 h at room temperature. DAPI was used as counterstain.

For IF on human tissue, frozen tissue sections were fixed in acetone for 10 min. Slides were blocked for 1 h in 3% BSA/PBS at room temperature, and incubated overnight at 4°C with primary antibodies for FAP (1:50; R&D Systems) and αSMA (1:200; Sigma-Aldrich). After washes, the slides were incubated for 1 h at room temperature with anti–mouse Alexa Fluor 488 (1:500; Invitrogen) and anti–sheep Alexa Fluor 555 (1:500; Invitrogen) secondary antibodies, then washed and mounted with Vectashield Hardset containing DAPI (Vector Laboratories). The use of human tissues in this study was approved by The Ethics Review Board (EPN) of Northern Sweden.

### Imaging

Live cell and immunofluorescence imaging of monocultures and co-cultures was done on a Perkin Elmer Ultraview Vox spinning disc confocal system (Waltham MA) using the Velocity 6.3 software. The system consisted of a Nikon Ti Eclipse inverted microscope (Morrell Instruments Melville NY), Yokogawa CSU-Xi spinning disk, Perkin Elmer laser module 2.0 (6 lines), Hamamatsu R2 CCD, motorized peizo Z stage from Applied Scientific Imaging (Eugene OR), environmental chamber from In Vivo Scientific, temperature regulation with the Smart Air-Therm heater from World Precision Instruments (Sarasota FL), atmosphere was provided using premixed hematology gas with 5% CO_2_.

Fluorescence imaging of fixed tissue was done with a Leica TCS SP8 laser scanning confocal (Boulder Grove Il), controlled by the LAS AF 3.3.10134 software. This confocal was mounted on a DMI 6000 CS inverted microscope, equipped with 4 laser lines and 2 PMTs. Bright field imaging of tissue slides were obtained with an Axio Imager.A2 (ZEISS).

Quantification of fluorescence intensity was performed with the Velocity 6.3 software.

### Electron microscopy

Co-cultures were processed for electron microscopy as previously described (Arun et al., 2016). In brief, co-cultures were fixed overnight with 2% glutaraldehyde and 2% PFA in PBS, rinsed in distilled water and post-fixed with 1% osmium tetroxide in 1.5% potassium ferrocyanide for 1 h. After dehydration, samples were resin infiltrated and polymerized overnight at 60°C. 100 nm thin sections were collected on 100 mesh grids with and without a supporting film. Following counterstaining with lead citrate, sections were examined in a Hitachi H-7000 transmission electron microscope operated at 75 kV. Images were recorded on Kodak 4489 film and scanned at 2400 dpi.

### Western blot

PSCs or organoids were harvested in Cell Recovery Solution (Corning) and incubated rotating for 1 h at 4°C. Cells were then pelleted, and lysed in 0.1% Triton X-100, 15 mM NaCl, 0.5 mM EDTA, 5 mM Tris, pH 7.5 supplemented with protease Mini-complete protease inhibitors (Roche) and a phosphatase inhibitor cocktail (PhosSTOP; Roche). Cells were incubated on ice for 30 min before clarification. Standard procedures were used for Western blot. In brief, protein lysates were separated by SDS-PAGE, transferred to a polyvinylidene difluoride (PVDF) membrane, blocked with 5% BSA in TBST (1% Tween 20, tris-buffered saline), and incubated with primary antibodies overnight at 4°C. Proteins were detected using HRP-conjugated secondary antibodies. Primary antibodies used were: PDGFRα (Cell Signaling Technology), PDGFRβ (Cell Signaling Technology), αSMA (Dako), Hsp90α (EMD Millipore), Actin (Cell Signaling Technology), Phospho-STAT3 (Cell Signaling Technology), STAT3 (Cell Signaling Technology), Pan-Actin (Cell Signaling Technology).

### Secretome analysis

Conditioned media was collected after incubation with monocultures of PSCs, CAFs, mouse or human organoids, co-cultures, or Matrigel-only controls for 3–4 d, filtered through a 0.45-µm filter to remove cell debris, and frozen at –20°C. Frozen conditioned media was then thawed and used for cytokine dot blots (R&D Systems) according to the manufacturer's instructions.

### ELISA

For ELISA of conditioned media of co-cultures or monocultures of PSCs, human CAFs, and mouse and human tumor organoids, cultures were grown in DMEM containing 5% FBS for 3–5 d. Media was then collected, filtered through a 0.45-µm filter to remove cell debris, aliquoted, and frozen at –20°C. Thawed media was assayed using the manufacturer's protocol. The following ELISA assays used were: IL-6 (R&D Systems), IL-11 (R&D Systems), and LIF (R&D Systems).

### Dot blot and Western blot quantification

Dot blots and Western blot films were scanned at 600 dpi and then quantified in ImageJ (National Institutes of Health). Scanned blots were background subtracted using a rolling ball radius of 50 pixels, and then inverted before being quantified using the integrated pixel density function. For Western blots, a rectangular region was used to quantify each band. Quantification is presented as pixel intensity of pSTAT3 normalized to total STAT3, loading control, and the control condition. For dot blots, a circular region was used to quantify each pair of dots, which was then averaged.

### Proliferation assays

GFP-expressing organoids and mCherry-expressing PSCs were dissociated into single cells and counted. Both cell types were mixed in a 1:1 ratio in 50% Matrigel and 50% media (DMEM with 5% FBS). 70 µl of a cell suspension containing 7,000 cells of each cell type was plated per well on black 96-well plates with clear bottoms (Corning) on ice. After 30-min incubation in a cell incubator, 200 µl prewarmed media containing 10.5 µM RhoK-inhibitor (Sigma-Aldrich) was added on top of the cells. Fluorescence intensity was measured for both GFP and mCherry once a day for up to 7 consecutive days, on a SpectaMax I3 (Molecular Devices) by scanning each well at 52 points and averaging the intensity.

When proliferation of nonfluorescent PSCs was measured, 5,000 cells were seeded in 96-well plates in 50% Matrigel/PBS and cultured in 150 µl control media or conditioned media. Cell proliferation was followed for 5 d with CellTiter-Glo (Promega), with measurements every 24 h.

### RNA seq

Samples were collected in 1 ml of TRIzol Reagent (Thermo Fisher Scientific) and stored at −80°C. RNA was extracted using the PureLink RNA mini kit (Thermo Fisher Scientific). RNA quality was assessed on a bioanalyzer using the Agilent RNA 600 Nano kit. We used poly-A pull-down to enrich for mRNAs from total RNA samples (0.2–1 µg per sample, RIN > 8) and proceeded to library preparation using Illumina TruSeq RNA prep kit. Libraries were then sequenced using Illumina HiSeq2000 at the Columbia Genome Center. We multiplexed samples in each lane, which yielded targeted number of single-end 100-bp reads for each sample, as a fraction of 180 million reads for the whole lane. We used RTA (Illumina) for base calling and bcl2fastq (version 1.8.4) for converting BCL to fastq format, coupled with adaptor trimming. We mapped the reads to a reference genome (Human, NCBI/build37.2; Mouse, UCSC/mm9) using Tophat (Trapnell et al., 2009; version 2.0.4) with 4 mismatches (–read-mismatches = 4) and 10 maximum multiple hits (–max-multihits = 10). To tackle the mapping issue of reads that are from exon–exon junctions, Tophat infers novel exon–exon junctions ab initio, and combined them with junctions from known mRNA sequences (refgenes) as the reference annotation. We estimated the relative abundance (i.e., expression level) of genes and splice isoforms using cufflinks (Trapnell et al., 2010; version 2.0.2) with default settings. All RNA-seq data are available at Gene Expression Omnibus (GEO) under the accession no. GSE93313.

### Differential expression analysis and GSEA

Genes expressed in fewer than two libraries were filtered out before differential expression testing. The principle component analysis was calculated using the prcomp function available in R and plotted using a customized R script. Expression normalization and differential expression testing were performed using DESeq (Anders and Huber, 2010), with dispersion estimation parameters set as: ‘method = “per-condition”, sharingMode = “maximum”, fitType = “parametric.”’ Genes with adjusted P < 0.01 were selected as significantly differentially expressed between conditions. All plots were produced using customized R scripts.

GSEA on the RNA-seq data were performed by entering fold change data from the differential expression analysis into the GSEA software (Broad Institute) using the Gene sets database c2.cp.v5.1.symbols.

### Statistics

For graphical representation of data and statistical analysis, GraphPad Prism was used. Statistical analysis was performed using Student’s *t* test. If multiple *t* tests of the same dataset were performed, correction for multiple comparisons was made using the Sidak-Bonferroni method. Data are presented as mean of biological replicates ± SD unless otherwise indicated.

### Online supplemental material

Fig. S1 shows the isolation process and validation data for the PSCs and human CAFs used in this study. Fig. S2 shows supplementary data from investigating fibroblast heterogeneity in the co-culture model system and the isolation process and validation data for the mouse KPC CAFs used in this study. Table S1, provided as an Excel file, contains the RNA expression analysis (DeSEQ) and pathway analysis (GSEA) comparing quiescent PSCs, iCAFs, and myofibroblasts. 

## References

[bib1] Anders S., and Huber W. 2010 Differential expression analysis for sequence count data. Genome Biol. 11:R106 10.1186/gb-2010-11-10-r106 20979621 PMC3218662

[bib2] Apte M.V., Haber P.S., Applegate T.L., Norton I.D., McCaughan G.W., Korsten M.A., Pirola R.C., and Wilson J.S. 1998 Periacinar stellate shaped cells in rat pancreas: identification, isolation, and culture. Gut. 43:128–133. 10.1136/gut.43.1.128 9771417 PMC1727174

[bib3] Apte M.V., Park S., Phillips P.A., Santucci N., Goldstein D., Kumar R.K., Ramm G.A., Buchler M., Friess H., McCarroll J.A., 2004 Desmoplastic reaction in pancreatic cancer: role of pancreatic stellate cells. Pancreas. 29:179–187. 10.1097/00006676-200410000-00002 15367883

[bib4] Apte M.V., Wilson J.S., Lugea A., and Pandol S.J. 2013 A starring role for stellate cells in the pancreatic cancer microenvironment. Gastroenterology. 144:1210–1219. 10.1053/j.gastro.2012.11.037 23622130 PMC3729446

[bib5] Arun G., Diermeier S., Akerman M., Chang K.C., Wilkinson J.E., Hearn S., Kim Y., MacLeod A.R., Krainer A.R., Norton L., 2016 Differentiation of mammary tumors and reduction in metastasis upon Malat1 lncRNA loss. Genes Dev. 30:34–51. 10.1101/gad.270959.115 26701265 PMC4701977

[bib6] Bachem M.G., Schünemann M., Ramadani M., Siech M., Beger H., Buck A., Zhou S., Schmid-Kotsas A., and Adler G. 2005 Pancreatic carcinoma cells induce fibrosis by stimulating proliferation and matrix synthesis of stellate cells. Gastroenterology. 128:907–921. 10.1053/j.gastro.2004.12.036 15825074

[bib7] Bijlsma M.F., and van Laarhoven H.W. 2015 The conflicting roles of tumor stroma in pancreatic cancer and their contribution to the failure of clinical trials: a systematic review and critical appraisal. Cancer Metastasis Rev. 34:97–114. 10.1007/s10555-014-9541-1 25566685

[bib8] Boj S.F., Hwang C.I., Baker L.A., Chio I.I., Engle D.D., Corbo V., Jager M., Ponz-Sarvise M., Tiriac H., Spector M.S., 2015 Organoid models of human and mouse ductal pancreatic cancer. Cell. 160:324–338. 10.1016/j.cell.2014.12.021 25557080 PMC4334572

[bib58] Business Wire 2012 Infinity reports update from phase 2 study of saridegib plus gemcitabine in patients with metastatic pancreatic cancer. http://www.businesswire.com/news/home/20120127005146/en/Infinity-Reports-Update-Phase-2-Study-Saridegib

[bib9] Coppé J.P., Desprez P.Y., Krtolica A., and Campisi J. 2010 The senescence-associated secretory phenotype: the dark side of tumor suppression. Annu. Rev. Pathol. 5:99–118. 10.1146/annurev-pathol-121808-102144 20078217 PMC4166495

[bib10] Corcoran R.B., Contino G., Deshpande V., Tzatsos A., Conrad C., Benes C.H., Levy D.E., Settleman J., Engelman J.A., and Bardeesy N. 2011 STAT3 plays a critical role in KRAS-induced pancreatic tumorigenesis. Cancer Res. 71:5020–5029. 10.1158/0008-5472.CAN-11-0908 21586612 PMC3693754

[bib11] Desmoulière A., Guyot C., and Gabbiani G. 2004 The stroma reaction myofibroblast: a key player in the control of tumor cell behavior. Int. J. Dev. Biol. 48:509–517. 10.1387/ijdb.041802ad 15349825

[bib12] Erez N., Truitt M., Olson P., Arron S.T., and Hanahan D. 2010 Cancer-Associated Fibroblasts Are Activated in Incipient Neoplasia to Orchestrate Tumor-Promoting Inflammation in an NF-kappaB-Dependent Manner. Cancer Cell. 17:135–147. 10.1016/j.ccr.2009.12.041 20138012

[bib13] Erkan M., Adler G., Apte M.V., Bachem M.G., Buchholz M., Detlefsen S., Esposito I., Friess H., Gress T.M., Habisch H.J., 2012 StellaTUM: current consensus and discussion on pancreatic stellate cell research. Gut. 61:172–178. 10.1136/gutjnl-2011-301220 22115911 PMC3245897

[bib14] Feig C., Jones J.O., Kraman M., Wells R.J., Deonarine A., Chan D.S., Connell C.M., Roberts E.W., Zhao Q., Caballero O.L., 2013 Targeting CXCL12 from FAP-expressing carcinoma-associated fibroblasts synergizes with anti-PD-L1 immunotherapy in pancreatic cancer. Proc. Natl. Acad. Sci. USA. 110:20212–20217. 10.1073/pnas.1320318110 24277834 PMC3864274

[bib15] Flint T.R., Janowitz T., Connell C.M., Roberts E.W., Denton A.E., Coll A.P., Jodrell D.I., and Fearon D.T. 2016 Tumor-Induced IL-6 Reprograms Host Metabolism to Suppress Anti-tumor Immunity. Cell Metab. 24:672–684. 10.1016/j.cmet.2016.10.010 27829137 PMC5106372

[bib16] Froeling F.E., Feig C., Chelala C., Dobson R., Mein C.E., Tuveson D.A., Clevers H., Hart I.R., and Kocher H.M. 2011 Retinoic acid-induced pancreatic stellate cell quiescence reduces paracrine Wnt-β-catenin signaling to slow tumor progression. Gastroenterology. 141:1486–1497: 1497.e1–1497.e14. 10.1053/j.gastro.2011.06.047 21704588

[bib17] Garrido-Laguna I., Uson M., Rajeshkumar N.V., Tan A.C., de Oliveira E., Karikari C., Villaroel M.C., Salomon A., Taylor G., Sharma R., 2011 Tumor engraftment in nude mice and enrichment in stroma-related gene pathways predict poor survival and resistance to gemcitabine in patients with pancreatic cancer. Clin. Cancer Res. 17:5793–5800. 10.1158/1078-0432.CCR-11-0341 21742805 PMC3210576

[bib18] Hingorani S.R., Wang L., Multani A.S., Combs C., Deramaudt T.B., Hruban R.H., Rustgi A.K., Chang S., and Tuveson D.A. 2005 Trp53R172H and KrasG12D cooperate to promote chromosomal instability and widely metastatic pancreatic ductal adenocarcinoma in mice. Cancer Cell. 7:469–483. 10.1016/j.ccr.2005.04.023 15894267

[bib19] Hingorani S.R., Harris W.P., Beck J.T., Berdov B.A., Wagner S.A., Pshevlotsky E.M., Tjulandin S.A., Gladkov O.A., Holcombe R.F., Korn R., 2016 Phase Ib Study of PEGylated Recombinant Human Hyaluronidase and Gemcitabine in Patients with Advanced Pancreatic Cancer. Clin. Cancer Res. 22:2848–2854. 10.1158/1078-0432.CCR-15-2010 26813359 PMC7787348

[bib20] Huch M., Bonfanti P., Boj S.F., Sato T., Loomans C.J., van de Wetering M., Sojoodi M., Li V.S., Schuijers J., Gracanin A., 2013 Unlimited in vitro expansion of adult bi-potent pancreas progenitors through the Lgr5/R-spondin axis. EMBO J. 32:2708–2721. 10.1038/emboj.2013.204 24045232 PMC3801438

[bib21] Hwang R.F., Moore T., Arumugam T., Ramachandran V., Amos K.D., Rivera A., Ji B., Evans D.B., and Logsdon C.D. 2008 Cancer-associated stromal fibroblasts promote pancreatic tumor progression. Cancer Res. 68:918–926. 10.1158/0008-5472.CAN-07-5714 18245495 PMC2519173

[bib22] Jacobetz M.A., Chan D.S., Neesse A., Bapiro T.E., Cook N., Frese K.K., Feig C., Nakagawa T., Caldwell M.E., Zecchini H.I., 2012 Hyaluronan impairs vascular function and drug delivery in a mouse model of pancreatic cancer. Gut. 10.1136/gutjnl-2012-302529 PMC3551211 22466618

[bib23] Jesnowski R., Fürst D., Ringel J., Chen Y., Schrödel A., Kleeff J., Kolb A., Schareck W.D., and Löhr M. 2005 Immortalization of pancreatic stellate cells as an in vitro model of pancreatic fibrosis: deactivation is induced by matrigel and N-acetylcysteine. Lab. Invest. 85:1276–1291. 10.1038/labinvest.3700329 16127427

[bib24] Kalluri R. 2016 The biology and function of fibroblasts in cancer. Nat. Rev. Cancer. 16:582–598. 10.1038/nrc.2016.73 27550820

[bib25] Kim E.J., Sahai V., Abel E.V., Griffith K.A., Greenson J.K., Takebe N., Khan G.N., Blau J.L., Craig R., Balis U.G., 2014 Pilot clinical trial of hedgehog pathway inhibitor GDC-0449 (vismodegib) in combination with gemcitabine in patients with metastatic pancreatic adenocarcinoma. Clin. Cancer Res. 20:5937–5945. 10.1158/1078-0432.CCR-14-1269 25278454 PMC4254161

[bib26] Lee J.J., Perera R.M., Wang H., Wu D.C., Liu X.S., Han S., Fitamant J., Jones P.D., Ghanta K.S., Kawano S., 2014 Stromal response to Hedgehog signaling restrains pancreatic cancer progression. Proc. Natl. Acad. Sci. USA. 111:E3091–E3100. 10.1073/pnas.1411679111 25024225 PMC4121834

[bib27] Lesina M., Kurkowski M.U., Ludes K., Rose-John S., Treiber M., Klöppel G., Yoshimura A., Reindl W., Sipos B., Akira S., 2011 Stat3/Socs3 activation by IL-6 transsignaling promotes progression of pancreatic intraepithelial neoplasia and development of pancreatic cancer. Cancer Cell. 19:456–469. 10.1016/j.ccr.2011.03.009 21481788

[bib28] Mace T.A., Shakya R., Pitarresi J.R., Swanson B., McQuinn C.W., Loftus S., Nordquist E., Cruz-Monserrate Z., Yu L., Young G., 2016 IL-6 and PD-L1 antibody blockade combination therapy reduces tumour progression in murine models of pancreatic cancer. Gut.:gutjnl-2016-311585 10.1136/gutjnl-2016-311585 PMC5406266 27797936

[bib29] Marusyk A., Tabassum D.P., Altrock P.M., Almendro V., Michor F., and Polyak K. 2014 Non-cell-autonomous driving of tumour growth supports sub-clonal heterogeneity. Nature. 514:54–58. 10.1038/nature13556 25079331 PMC4184961

[bib30] Moffitt R.A., Marayati R., Flate E.L., Volmar K.E., Loeza S.G., Hoadley K.A., Rashid N.U., Williams L.A., Eaton S.C., Chung A.H., 2015 Virtual microdissection identifies distinct tumor- and stroma-specific subtypes of pancreatic ductal adenocarcinoma. Nat. Genet. 47:1168–1178. 10.1038/ng.3398 26343385 PMC4912058

[bib31] Moir J.A., Mann J., and White S.A. 2015 The role of pancreatic stellate cells in pancreatic cancer. Surg. Oncol. 24:232–238. 10.1016/j.suronc.2015.05.002 26080604

[bib32] Nagathihalli N.S., Castellanos J.A., VanSaun M.N., Dai X., Ambrose M., Guo Q., Xiong Y., and Merchant N.B. 2016 Pancreatic stellate cell secreted IL-6 stimulates STAT3 dependent invasiveness of pancreatic intraepithelial neoplasia and cancer cells. Oncotarget. 7:65982–65992. 27602757 10.18632/oncotarget.11786 PMC5323208

[bib33] Öhlund D., Elyada E., and Tuveson D. 2014 Fibroblast heterogeneity in the cancer wound. J. Exp. Med. 211:1503–1523. 10.1084/jem.20140692 25071162 PMC4113948

[bib34] Olive K.P., Jacobetz M.A., Davidson C.J., Gopinathan A., McIntyre D., Honess D., Madhu B., Goldgraben M.A., Caldwell M.E., Allard D., 2009 Inhibition of Hedgehog signaling enhances delivery of chemotherapy in a mouse model of pancreatic cancer. Science. 324:1457–1461. 10.1126/science.1171362 19460966 PMC2998180

[bib35] Özdemir B.C., Pentcheva-Hoang T., Carstens J.L., Zheng X., Wu C.C., Simpson T.R., Laklai H., Sugimoto H., Kahlert C., Novitskiy S.V., 2014 Depletion of carcinoma-associated fibroblasts and fibrosis induces immunosuppression and accelerates pancreas cancer with reduced survival. Cancer Cell. 25:719–734. 10.1016/j.ccr.2014.04.005 24856586 PMC4180632

[bib36] Provenzano P.P., Cuevas C., Chang A.E., Goel V.K., Von Hoff D.D., and Hingorani S.R. 2012 Enzymatic targeting of the stroma ablates physical barriers to treatment of pancreatic ductal adenocarcinoma. Cancer Cell. 21:418–429. 10.1016/j.ccr.2012.01.007 22439937 PMC3371414

[bib37] Putoczki T.L., Thiem S., Loving A., Busuttil R.A., Wilson N.J., Ziegler P.K., Nguyen P.M., Preaudet A., Farid R., Edwards K.M., 2013 Interleukin-11 is the dominant IL-6 family cytokine during gastrointestinal tumorigenesis and can be targeted therapeutically. Cancer Cell. 24:257–271. 10.1016/j.ccr.2013.06.017 23948300

[bib38] Quante M., Tu S.P., Tomita H., Gonda T., Wang S.S., Takashi S., Baik G.H., Shibata W., Diprete B., Betz K.S., 2011 Bone marrow-derived myofibroblasts contribute to the mesenchymal stem cell niche and promote tumor growth. Cancer Cell. 19:257–272. 10.1016/j.ccr.2011.01.020 21316604 PMC3060401

[bib39] Rhim A.D., Mirek E.T., Aiello N.M., Maitra A., Bailey J.M., McAllister F., Reichert M., Beatty G.L., Rustgi A.K., Vonderheide R.H., 2012 EMT and dissemination precede pancreatic tumor formation. Cell. 148:349–361. 10.1016/j.cell.2011.11.025 22265420 PMC3266542

[bib40] Rhim A.D., Oberstein P.E., Thomas D.H., Mirek E.T., Palermo C.F., Sastra S.A., Dekleva E.N., Saunders T., Becerra C.P., Tattersall I.W., 2014 Stromal elements act to restrain, rather than support, pancreatic ductal adenocarcinoma. Cancer Cell. 25:735–747. 10.1016/j.ccr.2014.04.021 24856585 PMC4096698

[bib41] Sherman M.H., Yu R.T., Engle D.D., Ding N., Atkins A.R., Tiriac H., Collisson E.A., Connor F., Van Dyke T., Kozlov S., 2014 Vitamin D receptor-mediated stromal reprogramming suppresses pancreatitis and enhances pancreatic cancer therapy. Cell. 159:80–93. 10.1016/j.cell.2014.08.007 25259922 PMC4177038

[bib42] Shi J., Wang E., Milazzo J.P., Wang Z., Kinney J.B., and Vakoc C.R. 2015 Discovery of cancer drug targets by CRISPR-Cas9 screening of protein domains. Nat. Biotechnol. 33:661–667. 10.1038/nbt.3235 25961408 PMC4529991

[bib43] Siegel R.L., Miller K.D., and Jemal A. 2016 Cancer statistics, 2016. CA Cancer J. Clin. 66:7–30. 10.3322/caac.21332 26742998

[bib44] Sousa C.M., Biancur D.E., Wang X., Halbrook C.J., Sherman M.H., Zhang L., Kremer D., Hwang R.F., Witkiewicz A.K., Ying H., 2016 Pancreatic stellate cells support tumour metabolism through autophagic alanine secretion. Nature. 536:479–483. 10.1038/nature19084 27509858 PMC5228623

[bib45] Srinivas S., Watanabe T., Lin C.S., William C.M., Tanabe Y., Jessell T.M., and Costantini F. 2001 Cre reporter strains produced by targeted insertion of EYFP and ECFP into the ROSA26 locus. BMC Dev. Biol. 1:4 10.1186/1471-213X-1-4 11299042 PMC31338

[bib46] Straussman R., Morikawa T., Shee K., Barzily-Rokni M., Qian Z.R., Du J., Davis A., Mongare M.M., Gould J., Frederick D.T., 2012 Tumour micro-environment elicits innate resistance to RAF inhibitors through HGF secretion. Nature. 487:500–504. 10.1038/nature11183 22763439 PMC3711467

[bib47] Sugimoto H., Mundel T.M., Kieran M.W., and Kalluri R. 2006 Identification of fibroblast heterogeneity in the tumor microenvironment. Cancer Biol. Ther. 5:1640–1646. 10.4161/cbt.5.12.3354 17106243

[bib48] Taga T., and Kishimoto T. 1997 Gp130 and the interleukin-6 family of cytokines. Annu. Rev. Immunol. 15:797–819. 10.1146/annurev.immunol.15.1.797 9143707

[bib49] Talar-Wojnarowska R., Gasiorowska A., Smolarz B., Romanowicz-Makowska H., Kulig A., and Malecka-Panas E. 2009 Clinical significance of interleukin-6 (IL-6) gene polymorphism and IL-6 serum level in pancreatic adenocarcinoma and chronic pancreatitis. Dig. Dis. Sci. 54:683–689. 10.1007/s10620-008-0390-z 18661238

[bib50] Tian H., Callahan C.A., DuPree K.J., Darbonne W.C., Ahn C.P., Scales S.J., and de Sauvage F.J. 2009 Hedgehog signaling is restricted to the stromal compartment during pancreatic carcinogenesis. Proc. Natl. Acad. Sci. USA. 106:4254–4259. 10.1073/pnas.0813203106 19246386 PMC2647977

[bib51] Trapnell C., Pachter L., and Salzberg S.L. 2009 TopHat: discovering splice junctions with RNA-Seq. Bioinformatics. 25:1105–1111. 10.1093/bioinformatics/btp120 19289445 PMC2672628

[bib52] Trapnell C., Williams B.A., Pertea G., Mortazavi A., Kwan G., van Baren M.J., Salzberg S.L., Wold B.J., and Pachter L. 2010 Transcript assembly and quantification by RNA-Seq reveals unannotated transcripts and isoform switching during cell differentiation. Nat. Biotechnol. 28:511–515. 10.1038/nbt.1621 20436464 PMC3146043

[bib53] Vonlaufen A., Phillips P.A., Xu Z., Goldstein D., Pirola R.C., Wilson J.S., and Apte M.V. 2008 Pancreatic stellate cells and pancreatic cancer cells: an unholy alliance. Cancer Res. 68:7707–7710. 10.1158/0008-5472.CAN-08-1132 18829522

[bib54] Waghray M., Yalamanchili M., Dziubinski M., Zeinali M., Erkkinen M., Yang H., Schradle K.A., Urs S., Pasca Di Magliano M., Welling T.H., 2016 GM-CSF mediates mesenchymal-epithelial cross-talk in pancreatic cancer. Cancer Discov. 6:886–899. 10.1158/2159-8290.CD-15-0947 27184426 PMC5549011

[bib55] Xu Z., Vonlaufen A., Phillips P.A., Fiala-Beer E., Zhang X., Yang L., Biankin A.V., Goldstein D., Pirola R.C., Wilson J.S., and Apte M.V. 2010 Role of pancreatic stellate cells in pancreatic cancer metastasis. Am. J. Pathol. 177:2585–2596. 10.2353/ajpath.2010.090899 20934972 PMC2966814

[bib56] Yuzawa S., Kano M.R., Einama T., and Nishihara H. 2012 PDGFRβ expression in tumor stroma of pancreatic adenocarcinoma as a reliable prognostic marker. Med. Oncol. 29:2824–2830. 10.1007/s12032-012-0193-0 22403002

[bib57] Zhang Y., Yan W., Collins M.A., Bednar F., Rakshit S., Zetter B.R., Stanger B.Z., Chung I., Rhim A.D., and di Magliano M.P. 2013 Interleukin-6 is required for pancreatic cancer progression by promoting MAPK signaling activation and oxidative stress resistance. Cancer Res. 73:6359–6374. 10.1158/0008-5472.CAN-13-1558-T 24097820 PMC3831882

